# Copper(II) Cyclopeptides with High ROS-Mediated Cytotoxicity

**DOI:** 10.1021/acs.bioconjchem.4c00561

**Published:** 2025-03-10

**Authors:** Sonia Boga, David Bouzada, Roi Lopez-Blanco, Axel Sarmiento, Iria Salvadó, David Alvar Gil, José Brea, María Isabel Loza, Natalia Barreiro-Piñeiro, José Martínez-Costas, Silvia Mena, Gonzalo Guirado, Alice Santoro, Peter Faller, M. Eugenio Vázquez, Miguel Vázquez López

**Affiliations:** † Centro Singular de Investigación en Química Biolóxica e Materiais Moleculares (CiQUS), Departamento de Química Orgánica, 16780 Universidade de Santiago de Compostela, 15782 Santiago de Compostela, Spain; ‡ Centro Singular de Investigación en Química Biolóxica e Materiais Moleculares (CiQUS), Departamento de Química Inorgánica, Universidade de Santiago de Compostela, 15782 Santiago de Compostela, Spain; § Innopharma Drug Screening and Pharmacogenomics Platform. Center for Research in Molecular Medicine and Chronic Diseases (CiMUS). Department of Pharmacology, Pharmacy and Pharmaceutical Technology, Universidade de Santiago de Compostela, 15782 Santiago de Compostela, Spain; ∥ Health Research Institute of Santiago de Compostela, 15782 Santiago de Compostela, Spain; ⊥ Centro Singular de Investigación en Química Biolóxica e Materiais Moleculares (CiQUS), Departamento de Bioquímica e Bioloxía Molecular, Universidade de Santiago de Compostela, 15782 Santiago de Compostela, Spain; # Departament de Química, 16719 Universitat Autònoma de Barcelona, Bellaterra, 08193 Barcelona, Spain; ∇ Institut de Chimie (UMR 7177), 27083 University of Strasbourg—CNRS, 67081 Strasbourg, France; ○ Institut Universitaire de France (IUF), 75231 Paris, France

## Abstract

Cu­(II) coordination complexes are emerging as promising anticancer agents due to their ability to induce oxidative stress through reactive oxygen species (ROS) generation. In this study, we synthesized and characterized two novel Cu­(II) metallopeptide systems, **1**/Cu­(II) and **2**/Cu­(II), derived from the oligocationic bipyridyl cyclopeptides **1** and **2,** and designed to enhance the transport of Cu­(II) into cells and increase ROS levels. Spectroscopic and electrochemical analyses confirmed the formation of stable metallopeptide species in aqueous media. Inductively coupled plasma mass spectrometry (ICP-MS) studies demonstrated that both metallopeptides significantly increase intracellular Cu­(II) accumulation in NCI/ADR-RES cancer cells, highlighting their role as efficient Cu­(II) transporters. Additionally, ROS generation assays revealed that **1**/Cu­(II) induces a substantial increase in intracellular ROS levels, supporting the hypothesis of oxidative stress-induced cytotoxicity. Cell-viability assays further confirmed that both **1**/Cu­(II) and **2**/Cu­(II) exhibit strong anticancer activity in a number of cancer cell lines, with IC_50_ values significantly lower than those of their free cyclopeptide counterparts or Cu­(II) alone, showing an order of activity higher than that of cisplatin. Finally, molecular modeling studies provided further insights into the structural stability and coordination environment of Cu­(II) within the metallopeptide complexes. These findings suggest that these Cu­(II) cyclometallopeptide systems hold potential as novel metal-based therapeutic agents, leveraging Cu­(II) transport and ROS increase as key strategies for cancer treatment.

## Graphical abstract

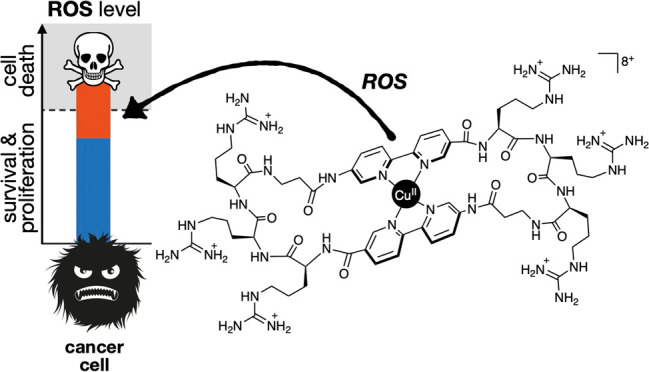

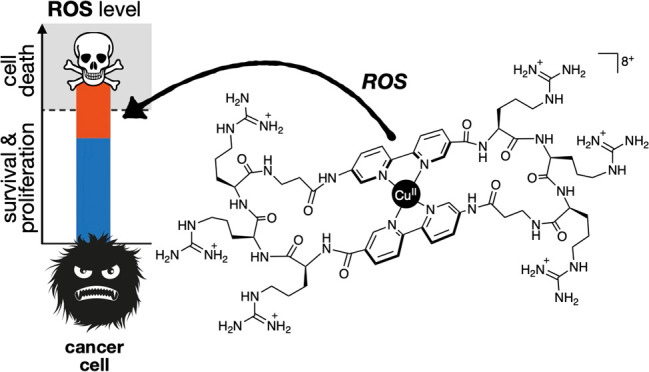

## Introduction

Recent high-throughput sequencing data have shown that thousands of different mutations can lead to cancer development and that the mutational signature of cancer is highly dynamic and might be different even between histopathologically identical tumors.^1,2^ These results suggest that anticancer strategies that target a single gene product will likely fail to deliver effective treatments and support the use of combination therapies.^3^ Unfortunately, most of the standard antitumorals converge on a small number of pathways, so new drugs are required for the design of combination therapies. Reactive oxygen species (ROS: ^1^O_2_, O_2_^•–^, HO^•^, H_2_O_2_) are a diverse class of radical species produced in all cells as a natural byproduct of metabolic processes that have essential functions in living organisms, such as signaling cell growth and differentiation, regulating enzymatic activity or inflammation processes.^4,5^ Growing evidence indicates that cancer cells have heightened levels of ROS that promote abnormal cell proliferation and diverse processes required for tumor progression.^6,7^ Such increased oxidative stress can also be toxic to the cells, causing lipid peroxidation, DNA damage, and protein oxidation, and makes tumoral cells more vulnerable to chemotherapeutic agents that further increase ROS generation or weaken the antioxidant defenses beyond levels compatible with cell survival.^8–10^ In this context, during the last 20 years, several copper­(II) coordination compounds, including complexes with 2,2′-bipyridine (Bpy) ligands, have been studied for their anticancer properties linked to their generation of reactive oxygen species.^11–15^ Among copper-based anticancer agents, Casiopeinas (a class of copper­(II) complexes containing a phenanthroline or a bipyridine ligand) have reached clinical trials in Mexico due to their potent cytotoxic activity and ROS-mediated mechanisms of action. These compounds exemplify the therapeutic potential of copper coordination complexes in cancer treatment and highlight the relevance of exploring alternative ligand scaffolds.^16^ On the other hand, some years ago, we reported a series of Bpy-based iridium­(III) cyclopeptides that display potent antitumoral activity comparable to that of cisplatin,^17^ and also that oligoarginine sequences can endow Bpy-based metallopeptides with cell-internalization capabilities.^18^ Based on these precedents, we envisioned that we could exploit the versatility of Bpy-derived peptide ligands to synthesize bioactive copper­(II) oligoarginine cyclopeptides that would display anticancer activity through induction of oxidative stress inside the cell.^19^

## Results and Discussion

### Design and Synthesis of Oligocationic Bipyridyl Cyclopeptides **1** and **2**

The solid-phase synthesis of the Bpy-derived oligocationic cyclopeptides first required the synthesis of a Fmoc-protected Bpy building block, Fmoc-βAlaBpy–OH (Scheme 1), which was obtained following previously reported procedures developed in our group.^17,20,21^ Having at hand this coordinating residue, we first assembled the precursor linear sequences using regular Fmoc solid-phase peptide synthesis (SPPS) on a chlorotrityl resin. Then, the linear, side chain-protected, free amine/carboxylate peptides, *H*-(βAlaBpy-[Arg­(Pbf)]_3_)_2_-COOH, and *H*-(βAlaBpy-[Arg­(Pbf)]_3_)_3_-COOH were detached from the solid support by treatment with a mild acidic mixture of AcOH/trifluoroethanol/CH_2_Cl_2_ (Scheme 1). These crude peptides were cyclized using the phosphonium coupling reagent PyAOP to avoid the formation of guanidino derivatives at the N-terminus,^22,23^ leading to the desired protected cyclopeptides. Removal of the Pbf groups in the Arg side chains with the standard trifluoroacetyl (TFA)/CH_2_Cl_2_/H_2_O/triisopropylsilane cocktail led to the final cyclopeptide ligands *cyclo*-(βAlaBpy-Arg_3_)_2_, **1**, and *cyclo*-(βAlaBpy-Arg_3_)_3_, **2**, which were purified by high-performance liquid chromatography (HPLC) (the purified samples were lyophilized and the cyclopeptides were obtained as TFA salts of the protonated Arg residues) and characterized by matrix-assisted laser desorption ionization-mass spectrometry (MALDI-MS) (see the Supporting Information).^17^

**Scheme 1:**
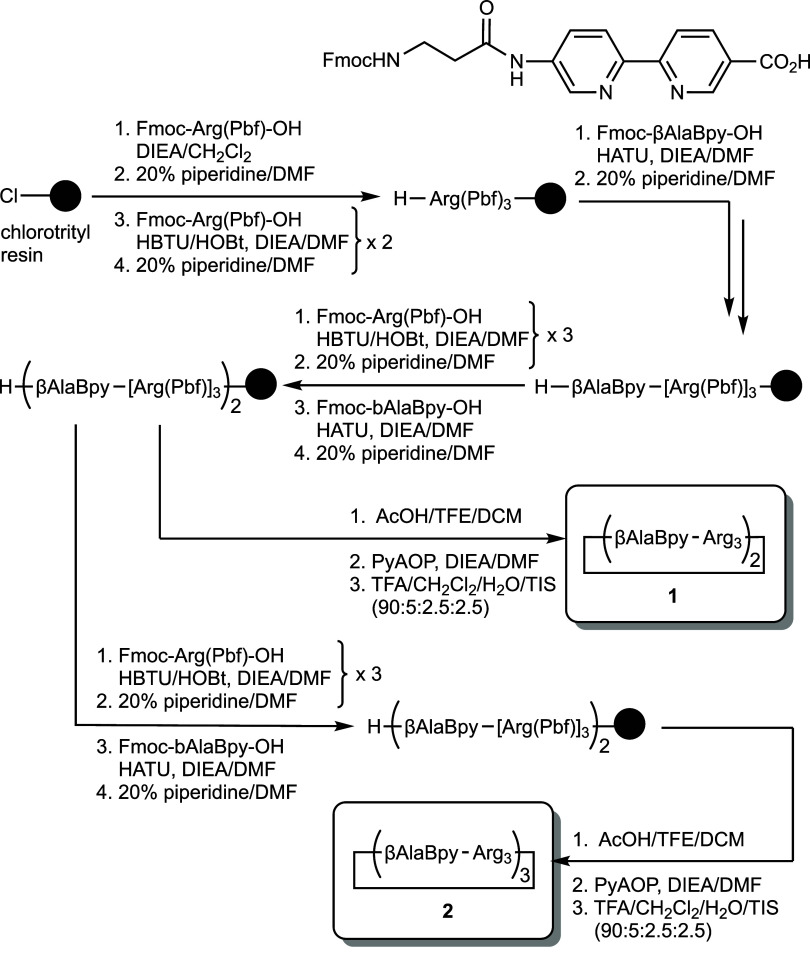
Solid-Phase Peptide Synthesis of Oligocationic Bipyridyl Cyclopeptides *cyclo*-(βAlaBpy-Arg_3_)_2_ (**1**) and *cyclo*-(βAlaBpy-Arg_3_)_3_ (**2**)^17^

### Synthesis and Characterization of the Metallopeptide Systems 1/Cu­(II) and 2/Cu­(II): Fluorescence Spectroscopy Studies

While the 2,2′-bipyridine ligand is known to be weakly emissive and for all practical purposes considered nonfluorescent,^24,25^ the 5′-amido-[2,2′-bipyridine]-5-carboxamide unit in the βAlaBpy building block is highly emissive, displaying an intense emission band at c.a. 410 nm with a quantum yield of 0.37.^21^ Furthermore, the fluorescence of the βAlaBpy units is quenched upon coordination to a wide range of divalent transition metal ions. We exploited this property to monitor the coordinative properties of cyclopeptides **1** and **2** in the presence of labile Cu­(II) ions in aqueous media. Thus, we measured the emission spectra of 2 μM solutions of the cyclopeptides **1** and **2** in 1 mM phosphate buffer and 10 mM NaCl, pH 6.5, upon excitation at 308 nm in the presence of increasing concentrations of Cu­(II) ions. The emission intensity profile of the titrations was fitted to different binding models depending on the cyclopeptide under study and according to the species identified in the MALDI spectra of the mixtures.^26^ Indeed, the MALDI spectra confirmed that cyclopeptides **1** and **2** assemble into various species with different stoichiometry that are in thermodynamic equilibrium in solution because the connection of the Bpy units within the cyclopeptides through their 5,5′ positions allows for both *exo* and *endo* coordination modes.^27^ Thus, the MALDI spectrum of the final titration mixture of the bisbipyridyl cyclopeptide **1** with Cu­(II) ions (see the Supporting Information), from now on, metallopeptide system **1**/Cu­(II), shows peaks consistent with the presence in solution of the 1:1 and 1:2 adducts (cyclopeptide:metal), so the titration was fit to a 1:2 model with a global dissociation constant *K*_D_ = 0.39 ± 0.19 μM using the DynaFit software (Figure 1a).^28,29^ On the other hand, the MALDI spectrum of trisbipyridyl cyclopeptide **2** with Cu­(II) ions (see the Supporting Information), from now on, metallopeptide system **2**/Cu­(II), shows peaks consistent with the 1:1, 1:2, and 1:3 adducts (cyclopeptide:metal), as well as peaks with higher masses involving two and even three cyclopeptide units. Due to the complexity of the mixture and the large errors associated with complex binding mechanisms involving many species, we characterized this complex using a simplified model, including 1:1 and 2:1 species, assuming that the higher-order species would be minor. Indeed, this model fits very well the experimental data (Figure 1b) with a global *K*_D_ = 0.24 ± 0.10 μM. The profiles of the fluorescence titrations for the two cyclopeptides clearly show that the quenching on the first Cu­(II) equivalent follows a linear trend and that this slows as the following Cu­(II) equivalents come into play. Therefore, the affinity of the first part of the titrations cannot be determined with this experiment as it is too strong. This behavior suggests that the first Cu­(II) ion coordinates more strongly to the cyclopeptides than the second or third, which can be explained by assuming that metallopeptide species in which Cu­(II) ions are coordinated to two Bpy units are thermodynamically favored, and that the coordination of successive metal ions to form species of higher nuclearity/order is disfavored. Owing to the high lability of Cu­(II) ions and the resulting highly dynamic equilibria in solution, our metallopeptides could not be observed by HPLC, which only showed the peaks corresponding to the free peptides.

**Figure 1:**
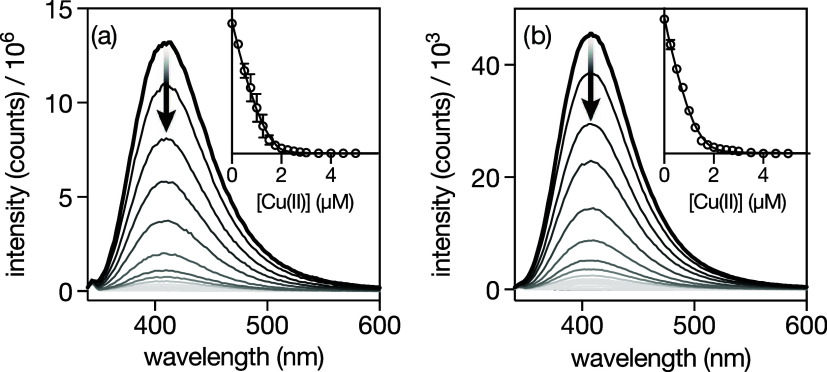
(a) Normalized emission spectrum of a 2 μM solution of cyclopeptide **1** in 1 mM phosphate buffer, 10 mM NaCl, pH 6.5, thick black line, and spectra of the same solution in the presence of increasing concentrations of Cu­(II) ions (progressively lighter shades of gray). Inset: titration profile showing the emission at 410 nm (λ_exc_ = 308 nm) of three independent fluorometric titrations and best fit according to the 1:2 model (cyclopeptide:metal) in DynaFit.^28,29^ (b) Same as in (a), but with cyclopeptide **2** and best fit according to a simplified model, including 1:1 and 2:1 species (see the text for details).

### UV–Vis Spectroscopy Studies

Incubation of low μM solutions of the cyclopeptides in phosphate buffer with excess of Cu­(II) ions (3 equiv for **1** and 5 equiv for **2**) resulted in clear hypo- and bathochromic shifts of the Bpy absorption band from c.a. 312 nm in the free cyclopeptides to c.a. 334 nm in the metallopeptide mixtures (Figure 2a,b), which supports the successful coordination of the Cu­(II) ion to the Bpy units. When using highly concentrated (500 μM) solutions of the cyclopeptides, we could observe a single broad band centered at c.a. 733 nm with extinction coefficients of 57 mol^–1^ L cm^–1^ for **1**/Cu­(II) and 114 mol^–1^ L cm^–1^ for **2**/Cu­(II), which can be assigned to d–d transitions.^30–34^ The shape, extinction coefficient, and position of these LF bands are consistent with the presence of [Cu­(Bpy)_2_]^2+^ coordination units in the mixtures,^35,36^ in agreement with the MALDI mass spectra and the fluorescence titration data.

We also performed UV–vis titrations of 6 μM solutions of the cyclopeptides **1** and **2** in phosphate buffer (1 mM, 10 mM NaCl, pH 6.5) with a stock solution of Cu­(II) ions in order to gain more information about the coordination processes that take place during the addition of the metal ions (Figures S4 and S5). In both cases, two different processes can be observed, one after the addition of approximately 1 equiv of Cu­(II) (Figures S4a and S5a, red line) and another one during the addition of the remaining equivalents until the end of the titration (Figures S4a and S5a, blue line). On the other hand, during the titration with the peptide ligand Ac-βAlaBpy-NH_2_ (Scheme S3), which consists only of the Bpy coordinative unit of both cyclopeptides, only a single coordinative process is observed, which ends when the L:M ratio is 2:1 (Figure S6). These data, taken together, suggest that for both cyclopeptides, the species formed during the first phase of the titrations is the 1:1 adduct (cyclopetide:metal) and it is during the addition of the remaining metal equivalents that higher-order minority coordinative species begin to form.

### EPR Studies

We performed electron paramagnetic resonance (EPR) studies at room temperature (300 K) on the metallopeptide system **1**/Cu­(II), formed by mixing 200 μM of cyclopeptide **1** and 3 equiv of Cu­(II) in 1 mM phosphate buffer 10 mM NaCl, pH 7.0, to characterize the coordinative environment of Cu­(II) ions in solution (Figure S7). The EPR spectrum of **1**/Cu­(II) is compatible with a Cu­(II) ion in a distorted square planar coordination with g//>g∧,^37^ a typical coordination geometry in d^9^ systems undergoing Jahn–Teller distortion.^35^

### CD Spectroscopy Studies

Following the initial spectroscopic characterization, we studied the cyclopeptides **1** and **2** and their corresponding Cu­(II) metallopeptide systems **1**/Cu­(II)—using 3 equiv Cu­(II)—and **2**/Cu­(II)—using 5 equiv Cu­(II)—by circular dichroism (CD) spectroscopy. Both cyclopeptides display very similar CD spectra characterized by an exciton band with a positive Cotton effect at ca. 318 nm that can be ascribed to the Bpy chromophores. The addition of Cu­(II) to the cyclopeptide solutions induced a bathochromic shift of this band to ca. 323 and ca. 354 nm, respectively, with a crossover at ca. 333 nm, plus an inversion of the Cotton effect.

Moreover, the Cu­(II) coordination induces the appearance of a new band with a positive sign centered at ca. 293 nm. The CD spectra of both Cu­(II) metallopeptide systems show very similar profiles, suggesting a similar arrangement of the Bpy chromophores around the Cu­(II) ions in both of them.^38^ This is in agreement with the results obtained in the MALDI mass spectra, as well as in the fluorescence and absorption studies, which suggest that for both cyclopeptides, the most favorable species are those in which a Cu­(II) ion is coordinated to two Bpy units (Figure 2c,d).

**Figure 2:**
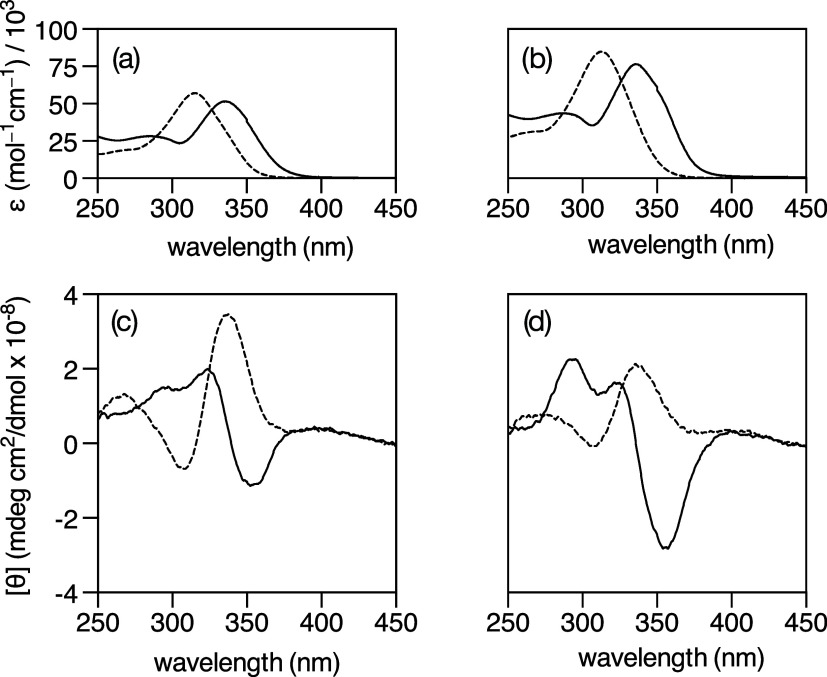
Top: UV–vis spectra of cyclopeptides **1** (a) and **2** (b), in 1 mM phosphate buffer, 10 mM NaCl, pH 6.5, before (dashed lines) and after (continuous lines) the addition of Cu­(II) ions (**1**/Cu­(II): 3 equiv; **2**/Cu­(II): 5 equiv). Bottom: CD spectra of 10 μM solutions of **1** (c) and **2** (d), in 1 mM phosphate buffer, 10 mM NaCl, pH 6.5, before (dashed lines) and after (continuous lines) the addition of Cu­(II) ions (**1**/Cu­(II): 3 equiv; **2**/Cu­(II): 5 equiv) in 1 mM phosphate buffer, 10 mM NaCl, pH 6.5. Spectra in parts b and c on the right are in the same scale as (a) in part b on the left.

### Molecular Modeling Studies

To confirm the proposed structure, density functional theory (DFT) calculations were performed using Gaussian 16, following a two-step optimization process. First, the complete structure of cyclopeptide **1** was optimized using the PM6 semiempirical method (Figure S18). To further refine the geometry, an ONIOM calculation was conducted, where PM6 was applied to the arginine residues, while the B3LYP method, incorporating Grimme’s Dispersion 3 (D3) correction, was used for the βAlaBpy residues with the 6-311+G­(d,p) basis set for H, N, C, and O atoms. In both steps, a solvation model based on density (SMD) for water was employed, and convergence was achieved with a tight (10^–5^) criterion. The most stable structure proposed for the **1**/Cu­(II) metallopeptide system, based on experimental data (Figures 3 and S19), was optimized using the same computational methodology just described for the analysis of the cyclopeptide and the Stuttgart/Dresden (SDD) pseudopotential was used for Cu­(II) (Figures 3).

**Figure 3:**
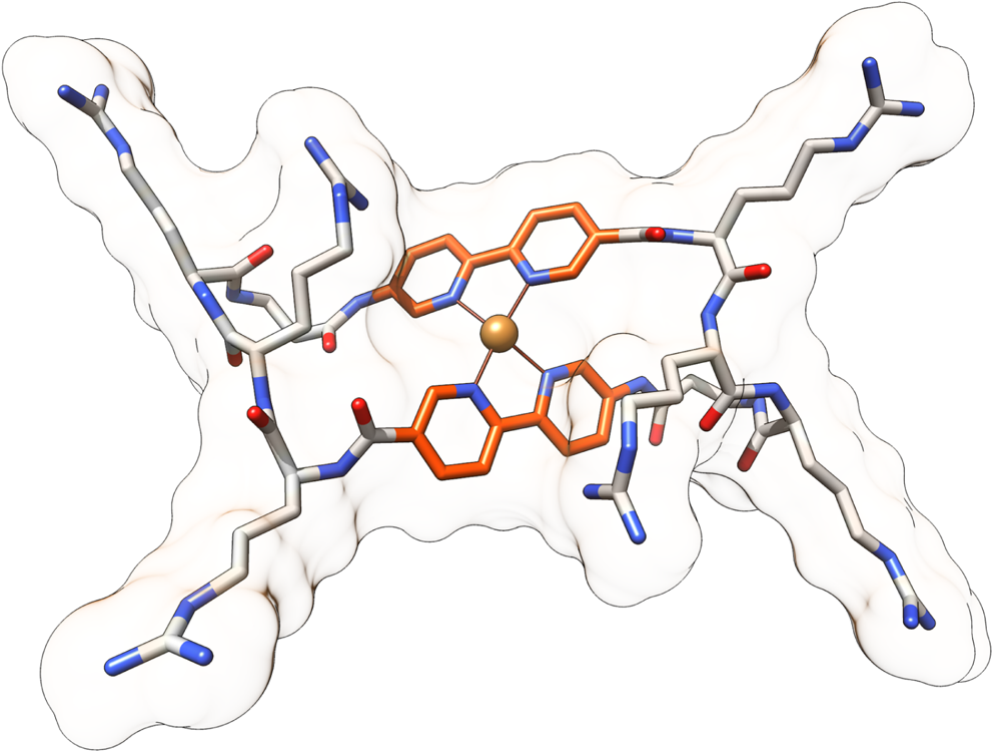
DFT-optimized model of the most stable structure of the **1**/Cu­(II) metallopeptide system based on experimental data.

### Cytotoxicity Studies

Having demonstrated the formation of the cyclopeptides **1** and **2**, we studied the cytotoxicity of these ligands and their corresponding Cu­(II) metallopeptide systems **1**/Cu­(II) (3 equiv of Cu^II^) and **2**/Cu­(II) (5 equiv of Cu^II^) with a set of tumor cell lines including HCT-116 (colon carcinoma), SF-268 (human glioma), NCI/ADR-RES (doxorubicin resistant ovarian carcinoma), and NCI-H460 (lung carcinoma), as well as in nontransformed lung fibroblast (MRC-5) (Table 1). Interestingly, the cytotoxicity of the free cyclopeptides seems to be dependent on the number of Bpy units, so the bisbipyridyl cyclopeptide **1** can be considered inactive in all of the cell lines under study, as the cell growth inhibition curve could not be completed, even reaching a concentration of 100 μM of compound. However, trisbipyridyl cyclopeptide **2** is moderately cytotoxic against all of the cell lines except HCT-116. On the other hand, both Cu­(II) metallopeptide systems show very high cytotoxicity against all of the cell lines under study, with very little differences among them. In particular, the IC_50_ values are in the same order as those of cisplatin for SF-268 and NCI-H460 and 1 order of magnitude higher for HCT-116 and NCI/ADR-RES. Interestingly, although the *E*_max_ values for **1**/Cu­(II) and **2**/Cu­(II) are fairly similar to that of cisplatin for HCT-116, SF-268, and NCI/ADR-RES cancer cell lines, as well as for the nontumoral MRC-5, they are significantly higher for NCI-H460. For instance, the measured *E*_max_ for cisplatin in this cancer cell line is 62%, whereas both Cu­(II) metallopeptide systems showed values of 96% under the same experimental conditions. IC_50_ is the concentration at which 50% growth inhibition is obtained and *E*_max_ represents the efficacy of the compound at its maximum concentration, expressed as the maximum % cell inhibition achieved by the compound (see Section S6 and Figure S8 of the Supporting Information).

**Table 1:** IC_50_ (μM) and *E*_max_ (%) Values of the Set of Cyclopeptides **1** and **2** and Their Corresponding Cu­(II) Metallopeptide Systems **1**/Cu­(II) and **2**/Cu­(II) for HCT-116, SF-268, NCI/ADR-RES, and NCI-H460 Tumoral Cell Lines, as well as for the Nontransformed Lung Fibroblast (MRC-5) Cell Line^a^

	HCT-116	SF-268	NCI/ADR-RES	NCI-H460	MRC-5
Cyclopeptides					
**1**	>100.0; 27 ± 1	>100.0; 45 ± 2	>100.0; 10 ± 2	>100.0; 32 ± 2	>100.0; 24 ± 2
**2**	>100.0; 80 ± 1	16.0 ± 1; 92 ± 1	28.0 ± 1; 86 ± 1	21.0 ± 1; 92 ± 1	18.0 ± 1; 92 ± 1
Cu(II) Metallopeptide Systems					
**1**/Cu(II)	2.81 ± 0.04; 94 ± 1	2.49 ± 0.03; 93 ± 1	2.42 ± 0.04; 88 ± 1	3.64 ± 0.07; 96 ± 1	1.73 ± 0.03; 92 ± 1
**2**/Cu(II)	2.82 ± 0.06; 95 ± 1	2.27 ± 0.07; 94 ± 2	2.83 ± 0.07; 88 ± 1	3.48 ± 0.02; 96 ± 1	1.48 ± 0.03; 92 ± 1
Controls					
cisplatin	13.0 ± 1; 94 ± 1	3.89 ± 0.08; 92 ± 1	13.0 ± 1; 86 ± 1	5.29 ± 0.46; 62 ± 4	5.70 ± 0.22; 92 ± 1
CuCl_2_·2H_2_O	>100.0; 94 ± 1	>100.0; 87 ± 1	99.0 ± 1; 91 ± 1	>100.0; 97 ± 1	79.0 ± 3; 91 ± 1

a IC_50_ is the concentration at which 50% growth inhibition is obtained and *E*_max_ represents the efficacy of the compound at its maximum concentration, expressed as the maximum % cell inhibition achieved by the compound. CuCl_2_ was selected as a negative control because, according to literature data, [Cu­(Bpy)_2_]^2+^ is not expected to be highly cytotoxic,^39^ unlike Cu­(II)-phen complexes.^40^

The metallopeptide systems **1**/Cu­(II) and **2**/Cu­(II), like cisplatin, did not show selective cytotoxicity in cancer cell lines compared to a noncancer cell line, MRC-5; this may be related to higher oxygen saturation levels in standard cell culture compared to in vivo conditions, leading to an altered redox balance in cultured noncancer cells,^41^ and does not mean that these metal complexes lack potential for the selective treatment of cancer.

### ICP-MS Studies

To evaluate the ability of the Cu­(II) metallopeptide systems to internalize in cancer cells, ICP-MS studies were performed in NCI/ADR-RES cells after 48 h of incubation with cyclopeptides **1** and **2**, as well as their corresponding **1**/Cu­(II) and **2**/Cu­(II) metallopeptide derivatives formed by mixing each cyclopeptide with 1 equiv Cu­(II), and using CuCl_2_·2H_2_O both as a Cu­(II) source and a control. The results confirm that both metallopeptide systems significantly enhance intracellular Cu­(II) accumulation, with **1**/Cu­(II) and **2**/Cu­(II) displaying comparable levels of Cu uptake, which exceed in both cases those observed for free cyclopeptides or Cu­(II) alone.

Interestingly, while free cyclopeptides **1** and **2** contribute to intracellular Cu­(II) accumulation, their effect is notably lower than that of the metallopeptide systems, suggesting that the coordination of Cu­(II) by the cyclopeptides plays a crucial role in facilitating metal transport across the cellular membrane, likely stabilizing Cu­(II) in a form more amenable to internalization. The observation that both cyclopeptides can transport Cu­(II) from the medium, where ICP-MS measurements detected 41.46 ng of Cu­(II) per 3 mL, further reinforces the idea that these molecules can act as copper carriers even in the absence of an exogenous Cu­(II) source. Moreover, the data indicate that free Cu­(II) (CuCl_2_·2H_2_O) by itself only results in a modest increase in intracellular copper, which suggests that passive diffusion of the metal ions is inefficient compared to the transport mediated by metal coordination.

To ensure consistency and avoid excess free Cu­(II), all experiments with metallopeptide systems were conducted by using equimolar amounts of cyclopeptide and Cu­(II). These findings highlight the role of metallopeptides as efficient Cu­(II) transporters, further supporting their potential biological relevance (Figure S9).

### Both Cu­(II) Metallopeptide Systems Efficiently Catalyze Ascorbate Oxidation

Having demonstrated the antitumoral activity and cell internalization properties of both Cu­(II) metallopeptide systems, we tested their ability to generate cytotoxic ROS in physiological media by measuring the rate of ascorbate oxidation. Indeed, the ascorbation rate has been related to the production of H_2_O_2_, which can be further reduced to HO^•^ (Scheme S4).^42,43^ The rate of ascorbate oxidation was monitored by variation in the absorption at 265 nm (100 μM ascorbate in 100 mM HEPES at pH 7.4) in the presence of Cu­(II) ions and the preformed Cu­(II) metallopeptide systems (added after 10 min incubation of Cu­(II) ions with cyclopeptides **1** or **2**) or the control ligand 5,5′-dimethylbipyridine (5DMB) (Figure 4).

**Figure 4:**
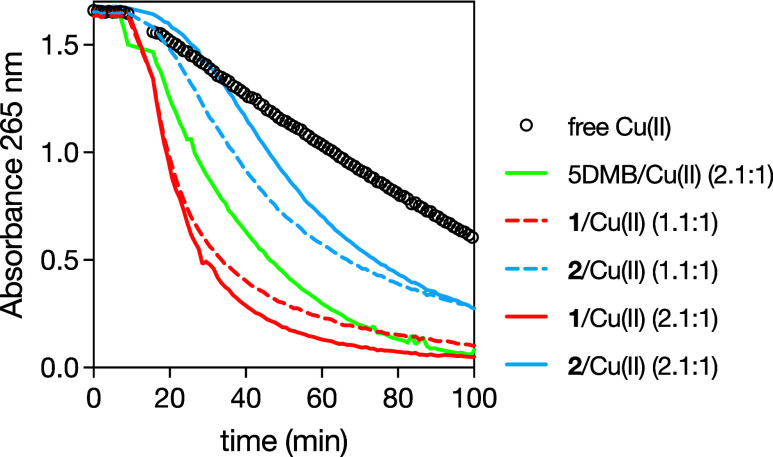
Time course of ascorbate oxidation monitored by the absorbance at 265 nm. The reaction was started by the addition of free Cu­(II) (circles) ions or the preformed Cu­(II) metallopeptide system to a solution of ascorbate in 100 mM HEPES at pH 7.4 after 10 min. The equivalents of each ligand (cyclopeptide or 5DMB) in the medium were fixed to 1.1 (dashed lines) or 2.1 (solid lines) with respect to the concentration of Cu­(II) ions. The final concentration of Cu­(II) ions and ascorbate were 300 nM and 100 μM, respectively.

The absorbance at 265 nm in the presence of free Cu­(II) ions changes linearly with time, whereas 5DMB/Cu­(II) follows classical kinetics.^44^ Both **1**/Cu­(II) and **2**/Cu­(II) were more active than Cu­(II) ions in buffer, a feature quite rarely observed as most copper complexes show lower rates of ascorbate oxidation than Cu­(II) ions.^45^ **1**/Cu­(II) had quite fast rate similar to 5DMB/Cu­(II). In contrast, **2**/Cu­(II) was only faster after a lag phase. **1**/Cu­(II) showed a more classical exponential decay but slowed down with time. This behavior could be explained by the degradation of the cyclopeptide in the presence of ROS (most likely via HO^•^). An indication for that is that the activity of the **1**/Cu­(II) complex is less slowed down with time with 2.1 equiv compared to 1.1. With an excess of ligand in 2.1, the Cu­(II) ions can coordinate undegraded cyclopeptide **1** and continue their catalytic action for a longer period of time.

In contrast, the **2**/Cu­(II) complex shows a lag phase at the beginning of the experiment, which could be explained by a rearrangement process between a resting state and a catalytically active state. At this point, it should be noted that at the beginning of the experiment, the copper ions are all in an oxidation state +2 and then must cycle between Cu­(II)/Cu­(I). **2**/Cu­(II) is slower than **1**/Cu­(II) but shows the same behavior in terms of the dependence of its activity on its concentration in the medium. At 10 μM, **1**/Cu­(II), and **2**/Cu­(II) become much faster than 5DMB/Cu­(II), according to the decrease in the absorbance band at 265 nm of 5DMB/Cu­(II) (Figures S12 and S13). However, for both Cu­(II) metallopeptide systems, this occurs almost immediately. A reasonable hypothesis for this behavior is that higher concentrations favor the formation of more active dinuclear and higher-order complexes. Interestingly, we observed a new absorption band at 390 nm for **1**/Cu­(II) and **2**/Cu­(II) but not for 5DMB/Cu­(II), which strongly indicates that the species present in the medium at the end of the experiments are not the same as those at the beginning (Figure S14). Finally, it should be noted that there is no correlation between the ascorbate oxidation activity and cell cytotoxicity (Table 1). Specifically, both metallopeptide systems show high and very similar cytotoxicity against all of the cell lines studied, but **1**/Cu­(II) is more active than **2**/Cu­(II) against ascorbate oxidation. However, note that cyclopeptide **2** shows some cytotoxicity in the free state against all but one of the cell lines studied, which is not observed in the case of cyclopeptide **1**.

### Intracellular ROS Generation Induced by the **1**/Cu­(II) Metallopeptide System

Measurement of intracellular ROS levels in NCI/ADR-RES cells treated with Cu­(II) ions, cyclopeptide **1**, or the **1**/Cu­(II) metallopeptide system confirmed a time-dependent increase in the level of ROS generation. In short, after 24 h of incubation, cells were stained with ROS Assay Stain 1× and treated with 100 μM Cu­(II) (CuCl_2_·2H_2_O), cyclopeptide **1**, or **1**/Cu­(II), along with 125 μM H_2_O_2_, and fluorescence was recorded (λ_ex_ = 495/λ_em_ = 520 nm) every hour for the first 5 h and again at 24 h. The experiment with the metallopeptide system was conducted by using equimolar amounts of cyclopeptide **1** and Cu­(II) to prevent an excess of free copper in the medium. The results show that **1**/Cu­(II) induces the highest ROS levels, particularly at 24 h, while cyclopeptide **1** also promotes ROS generation, albeit to a lesser extent (Figure S15). Cu­(II) ions alone has a moderate effect, and untreated cells exhibit minimal fluorescence. Considering these findings alongside ICP-MS data, which demonstrate that **1**/Cu­(II) efficiently increases intracellular Cu­(II) levels and that trace Cu­(II) present in the culture medium can be internalized by free cyclopeptide **1**, these results reinforce the proposed mechanism of action. The cytotoxicity of the metallopeptide systems appears to be closely linked to their ability to enhance intracellular oxidative stress via increased ROS production, likely driven by Cu­(II) accumulation within the cells.

### Electrochemistry of the Cu­(II) Metallopeptide Systems in Water

Following the demonstration of the capability of the metallopeptide systems **1**/Cu­(II) and **2**/Cu­(II) to generate ROS in water media and considering that these reactions are only possible due to the ability of their copper centers to carry out redox processes between their +2 and +1 oxidation states, we decided to study their electrochemical properties by cyclic voltammetry.

The electrochemical behaviors of cyclopeptides **1** and **2** in solution are very similar in all cases. A single irreversible reduction wave is observed at c.a. *E*_pc_ = −0.9 V (vs SCE) (Figure S11a–d). This reduction wave can be attributed to Bpy moieties of the cyclopeptide structures by comparison with the electrochemical behavior of 2,2′-bipyridine pure solutions under the same conditions (Figure S11e,f). As the scan rate (v, V s^–1^) is increased, the wave becomes reversible, indicating the stability of the reduced peptide at that pH. A more detailed analysis of the electron transfer indicates that the electron transfer is fast (peak width of 60 mV). However, the electrochemical behavior of the metallopeptide systems **1**/Cu­(II)—3 equiv of Cu­(II)—and **2**/Cu­(II)—5 equiv of Cu­(II)—in solution are quite different. Figure 5a shows the cyclic voltammetry response for cyclopeptide **1** and its metallopeptide system **1**/Cu­(II), which are relatively complex due to the overlap with the redox processes of the uncomplexed Cu­(II) ions in solution. Note that the electrochemical signals related to the uncoordinated Cu­(II) ions (asterisks) can be assigned by comparison with the electrochemical data obtained for pure CuCl_2_ solutions under the same experimental conditions (Figure S10). Thus, starting from a cathodic scan, two reduction electronic transfers from Cu­(II) to Cu­(I) can be distinguished at *E*_pc1,**1**/Cu(II)_ = −0.21 V and *E*_pc2,**1**/Cu(II)_ = −0.54 V, which indicate the presence of two Cu­(II) ions per cyclopeptide. In the oxidation sweep, a single anion peak is observed corresponding to the oxidation of Cu­(I) to Cu­(II) at *E*_pa,**1**/Cu(II)_ = 0.34 V. On the other hand, Figure 5b shows the CV response of cyclopeptide **2** and its metallopeptide system, **2**/Cu­(II), as a single electronic transfer of reduction from Cu­(II) to Cu­(I) at *E*_pc,**2**/Cu(II)_ = −0.57 V, which would indicate the presence of a single Cu­(II) ion per cyclopeptide. The oxidation scan also shows a single peak corresponding to the process of oxidation of Cu­(I) to Cu­(II) at *E*_pa,**2**/Cu(II)_ = 0.34 V. Again, the fact that the oxidation wave is irreversible suggests conformational or coordination changes from Cu­(I) to Cu­(II). The oxidation potentials from Cu­(I) to Cu­(II) for both cyclopeptide systems are the same, indicating that the coordinative environment of the Cu­(II) ions is similar in both cases. As noted above, the fact that the oxidation waves are irreversible indicates conformational and/or coordination changes between the oxidized and reduced forms of the metal. Finally, the reduction potentials from Cu­(II) to Cu­(I) are different depending on the cyclopeptide. This could suggest that OH/H_2_O bridges may exist in one of the two systems when the metal is in its reduced form.

**Figure 5:**
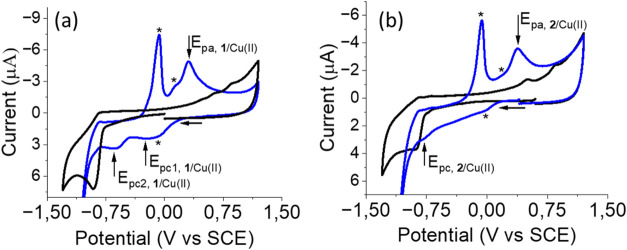
Cyclic voltammetry of 1 mM Cu­(II) metallopeptide systems **1**/Cu­(II) (3 equiv of Cu^II^) and **2**/Cu­(II) (5 equiv of Cu^II^) in an aqueous solution of 50 mM AMPD/HCl pH 5 + 0.1 M NaClO_4_, on a glassy carbon disk (diameter 1 mm). Scan rate 0.5 V·s^–1^. (a) Cyclopeptide **1** (black line) and metallopeptide system **1**/Cu­(II) (blue line); (b) cyclopeptide **2** (black line) and metallopeptide system **2**/Cu­(II) (blue line).

## Conclusions

This study demonstrates that the novel oligocationic bipyridyl cyclopeptides **1** and **2** effectively coordinate Cu­(II) ions, forming the metallopeptide systems **1**/Cu­(II) and **2**/Cu­(II). These metallopeptide systems significantly enhance the intracellular accumulation of Cu­(II) ions in NCI/ADR-RES cancer cells, as confirmed by ICP-MS analysis. Both Cu­(II) metallopeptide systems exhibit comparable Cu­(II) uptake efficiency, surpassing that of the free cyclopeptides or Cu­(II) alone, suggesting that metallopeptide coordination facilitates the transport of Cu­(II) across the cell membrane. ROS generation assays showed that **1**/Cu­(II) induces a significant increase in intracellular ROS levels, reinforcing the role of oxidative stress in its cytotoxic mechanism, while cyclopeptide **1** alone also promotes ROS production but to a lesser extent. Cytotoxicity assays demonstrated that both **1**/Cu­(II) and **2**/Cu­(II) exhibit potent cytotoxic activity in a number of cancer cell lines, with significantly lower IC_50_ values compared to the free cyclopeptides or Cu­(II) alone, showing an order of activity higher than that of cisplatin. Finally, molecular modeling studies provided valuable insights into the coordination environment and stability of the metallopeptide systems, further supporting their structural integrity in biological media. These findings highlight the potential of these Cu­(II) metallopeptide systems as promising candidates for metal-based anticancer strategies, where the ability to increase intracellular Cu­(II) levels, enhance ROS production, and induce cytotoxicity could be key factors in their therapeutic activity.

## Experimental Procedures

All of the experimental procedures are described in detail in the Supporting Information.

## Supplementary Material



## Data Availability

The data supporting this article have been included as part of the Supporting Information.

## Notes

Comprehensive overview of the experimental procedures and additional data supporting this study; details on author contributions, as well as sections covering the synthetic reagents used and the synthesis, purification, and characterization of cyclopeptides **1** and **2**, including MALDI characterization; the synthesis, purification, and characterization of Ac-βAlaBpy-NH_2_, along with studies on its Cu­(II) binding behavior, as well as that of cyclopeptides **1** and **2** in aqueous media; cytotoxicity studies, ICP studies, electrochemical studies, and ROS studies; the proposed most stable structures for the metallopeptide systems, based on experimental data, are presented, along with molecular modeling studies providing additional structural insights (PDF)

## References

[1] Marusyk A., Almendro V., Polyak K. (2012). Intra-tumour heterogeneity: a looking
glass for cancer?. Nat. Rev. Cancer.

[2] Stratton M. R., Campbell P. J., Futreal P. A. (2009). The cancer
genome. Nature.

[3] Bock C., Lengauer T. (2012). Managing drug resistance
in cancer: lessons from HIV
therapy. Nat. Rev. Cancer.

[4] Dickinson B. C., Chang C. J. (2011). Chemistry and biology
of reactive oxygen species in
signaling or stress responses. Nat. Chem. Biol..

[5] Adams L., Franco M. C., Estevez A. G. (2015). Reactive
nitrogen species in cellular
signaling. Exp. Biol. Med..

[6] Schumacker P. T. (2006). Reactive
oxygen species in cancer cells: live by the sword, die by the sword. Cancer Cell.

[7] Cheung E. C., Vousden K. H. (2022). The role of ROS in tumour development
and progression. Nat. Rev. Cancer.

[8] Trachootham D., Alexandre J., Huang P. (2009). Targeting cancer cells by ROS-mediated
mechanisms: a radical therapeutic approach?. Nat. Rev. Drug Discovery.

[9] Li L., Ishdorj G., Gibson S. B. (2012). Reactive oxygen species regulation
of autophagy in cancer: implications for cancer treatment. Free Radic. Biol. Med..

[10] Gorrini C., Harris I. S., Mak T. W. (2013). Modulation
of oxidative stress as
an anticancer strategy. Nat. Rev. Drug Discovery.

[11] Jomova K., Valko M. (2011). Advances in metal-induced
oxidative stress and human disease. Toxicology.

[12] Butcher K., Kannappan V., Kilari R. S., Morris M. R., McConville C., Armesilla A. L., Wang W. (2018). Investigation of the key chemical
structures involved in the anticancer activity of disulfiram in A549
non-small cell lung cancer cell line. BMC Cancer.

[13] Ng C. H., Kong S. M., Tiong Y. L., Maah M. J., Sukram N., Ahmad M., Khoo A. S. B. (2014). Selective
anticancer copper­(II)-mixed
ligand complexes: targeting of both ROS and proteasome. Metallomics.

[14] Hussain A., AlAjmi M. F., Rehman M. T., Amir S., Husain F. M., Alsalme A., Siddiqui M. A., AlKhedhairy A. A., Khan R. A. (2019). Copper­(II) complexes as potential anticancer and Nonsteroidal
anti-inflammatory agents: In vitro and in vivo studies. Sci. Rep..

[15] Santini C., Pellei M., Gandin V., Porchia M., Tisato F., Marzano C. (2014). Advances in copper
complexes as anticancer agents. Chem. Rev..

[16] Aguilar-Jiménez Z., Espinoza-Guillén A., Resendiz-Acevedo K., Fuentes-Noriega I., Mejía C., Ruiz-Azuara L. (2023). The importance
of being casiopeina as polypharmacologycal
profile (mixed chelate–copper (II) complexes and their in vitro
and in vivo activities). Inorganics.

[17] Salvadó I., Gamba I., Montenegro J., Martínez-Costas J., Brea J. M., Loza M. I., Vázquez López M., Vázquez M. E. (2016). Membrane-disrupting
iridium­(iii) oligocationic organometallopeptides. Chem. Commun..

[18] Gamba I., Salvadó I., Rama G., Bertazzon M., Sánchez M. I., Sánchez-Pedregal V. M., Martínez-Costas J. M., Brissos R. F., Gamez P., Mascareñas J. L., Vázquez López M., Vázquez M. E. (2013). Custom-fit
ruthenium­(II) metallopeptides: a new twist to DNA binding with coordination
compounds. Chem. - Eur. J..

[19] Alcalde-Ordóñez A., Barreiro-Piñeiro N., McGorman B., Gómez-González J., Bpizada D., Rivadulla F., Vázquez M. E., Kellett A., Martínez-Costas J., López M. V. (2023). A copper­(ii)
peptide helicate selectively cleaves DNA replication foci in mammalian
cells. Chem. Sci..

[20] Gómez-González J., Bouzada D., Pérez-Márquez L. A., Sciortino G., Maréchal J.-D., Vázquez López M., Vázquez M. E. (2021). Stereoselective Self-Assembly of DNA Binding Helicates
Directed by the Viral β-Annulus Trimeric Peptide Motif. Bioconjugate Chem..

[21] Gómez-González J., Pérez Y., Sciortino G., Roldan-Martín L., Martínez-Costas J., Maréchal J.-D., Alfonso I., López M. V., Vázquez M. E. (2021). Dynamic
Stereoselection of Peptide Helicates and Their Selective Labeling
of DNA Replication Foci in Cells. Angew. Chem.,
Int. Ed..

[22] Albericio F., Bofill J. M., El-Faham A., Kates S. A. (1998). Use of Onium Salt-Based
Coupling Reagents in Peptide Synthesis. J. Org.
Chem..

[23] Marder O., Albericio F. (2003). Industrial
application of coupling reagents in peptide. Chim. Oggi.

[24] Dhanya S., Bhattacharyya P. K. (1992). Fluorescence behaviour of 2,2′-bipyridine in
aqueous solution. J. Photochem. Photobiol.,
A.

[25] Yagi M., Kaneshima T., Wada Y., Takemura K., Yokoyama Y. (1994). The effects
of conformation and coordination to zinc­(II) ions on the luminescence
properties of 2,2′-bipyridine, methyl-substituted 2,2′-bipyridines
and 2,2′-biquinoline. J. Photochem. Photobiol.,
A.

[26] McBryde, W. A. E. A Critical Review of Equilibrium Data for Proton and Metal Complexes of 1,10–phenanthroline, 2,2′–Bipyridyl and Related Compounds; Elsevier, 1978; pp 1–17.

[27] Tian L.-L., Wang C., Dawn S., Smith M. D., Krause J. A., Shimizu L. S. (2009). Macrocycles with
Switchable exo/endo Metal Binding
Sites. J. Am. Chem. Soc..

[28] Kuzmič P. (1996). Program DYNAFIT
for the analysis of enzyme kinetic data: application to HIV proteinase. Anal. Biochem..

[29] Kuzmič P. (2009). DynaFit-A
software package for enzymology. Methods Enzymol..

[30] Hathaway B. J., Billing D. E. (1970). The electronic properties
and stereochemistry of mono-nuclear
complexes of the copper­(II) ion. Coord. Chem.
Rev..

[31] Sacconi L., Ciampolini M. (1964). Pseudo-tetrahedral structure of some α-branched
copper­(II) chelates with Schiff bases. J. Chem.
Soc..

[32] Arena G., Bonomo R. P., Contino A., Sgarlata C., Spoto G., Tabbi G. (2004). Influence of the coordination geometry on the physicochemical properties
of a copper­(II) complex with a tailor-made calixarene-based ligand
bearing dipyridyl pendants. An ESR, UV-Vis and CV study. Dalton Trans..

[33] Amendola V., Miljkovic A., Legnani L., Toma L., Dondi D., Lazzaroni S. (2018). Self-Assembly
of Pseudorotaxane Structures from a Dicopper­(II)
Molecular Cage and Dicarboxylate Axles. Inorg.
Chem..

[34] Cárdenas D. J., Livoreil A., Sauvage J.-P. (1996). Redox Control of the Ring-Gliding
Motion in a Cu-Complexed Catenane: A Process Involving Three Distinct
Geometries. J. Am. Chem. Soc..

[35] Garribba E., Micera G., Sanna D., Strinna-Erre L. (2000). The Cu­(II)-2,2′-bipyridine system revisited. Inorg. Chim. Acta.

[36] Ozutsumi K., Kawashima T. (1991). Structure
of copper­(II)-bpy and -phen complexes: EXAFS
and spectrophotometric studies on the structure of copper­(II) complexes
with 2,2′-bipyridine and 1,10-phenanthroline in aqueous solution. Inorg. Chim. Acta.

[37] Ottaviani M. F., Bossmann S., Turro N. J., Tomalia D. A. (1994). Characterization
of starburst dendrimers by the EPR technique. 1. Copper complexes
in water solution. J. Am. Chem. Soc..

[38] Gamba I., Rama G., Ortega-Carrasco E., Berardozzi R., Sánchez-Pedregal V. M., Di Bari L., Maréchal J.-D., Vázquez M. E., Vázquez López M. (2016). The folding
of a metallopeptide. Dalton Trans..

[39] Shi Y., Toms B. B., Dixit N., Kumari N., Mishra L., Goodisman J., Dabrowiak J. C. (2010). Cytotoxicity of Cu­(II) and Zn­(II)
2,2′-Bipyridyl Complexes: Dependence of IC50 on Recovery Time. Chem. Res. Toxicol..

[40] Masuri S., Vaňhara P., Cabiddu M. G., Moráň L., Havel J., Cadoni E., Pivetta T. (2022). Copper­(II) Phenanthroline-Based
Complexes as Potential AntiCancer Drugs: A Walkthrough on the Mechanisms
of Action. Molecules.

[41] Stuart J. A., Fonseca J., Moradi F., Cunningham C., Seliman B., Worsfold C. R., Dolan S., Abando J., Maddalena L. A. (2018). How Supraphysiological Oxygen Levels
in Standard Cell
Culture Affect Oxygen-Consuming Reactions. Oxid.
Med. Cell. Longevity.

[42] Noël S., Perez F., Pedersen J. T., Alies B., Ladeira S., Sayen S., Guillon E., Gras E., Hureau C. (2012). A new water-soluble
Cu­(II) chelator that retrieves Cu from Cu­(amyloid-β) species,
stops associated ROS production and prevents Cu­(II)-induced Aβ
aggregation. J. Inorg. Biochem..

[43] Padayatty S. J., Levine M. (2016). Vitamin C: the known
and the unknown and Goldilocks. Oral Dis..

[44] Santoro A., Calvo J. S., Peris-Díaz M.
D., Krężel A., Meloni G., Faller P. (2020). The glutathione/metallothionein system
challenges the design of efficient O_2_-activating Cu-complexes. Angew. Chem., Int. Ed..

[45] Chassaing S., Collin F., Dorlet P., Gout J., Hureau C., Faller P. (2013). Copper and heme-mediated Abeta toxicity: redox chemistry,
Abeta oxidations and anti-ROS compounds. Curr.
Top. Med. Chem..

