# Supplementary material for: Copper(II) Cyclopeptides with High ROS-Mediated Cytotoxicity
Source: Bioconjug Chem. 2025 Mar 10;36(3):500–9. doi: 10.1021/acs.bioconjchem.4c00561 (PMC12128024; doi:10.1021/acs.bioconjchem.4c00561)
Supplement: Supplementary file 1 [file bc4c00561_si_001.pdf]

Supporting Information

Copper(II) cyclopeptides with high ROS-mediated cytotoxicity

Sonia Boga,[a†] David Bouzada,[a†] Roi Lopez-Blanco,[a] Axel Sarmiento,[b] Iria Salvadó,[b] David Alvar Gil,[b] José Brea,[c,d] María Isabel Loza,[c,d] Natalia Barreiro-Piñeiro,[e], José Martínez-Costas,[e] Silvia Mena,[f] Gonzalo Guirado,[f] Alice Santoro,[g] Peter Faller,[g,h] M. Eugenio Vázquez[a] and Miguel Vázquez López[b,*]

[a] *Centro Singular de Investigación en Química Biolóxica e Materiais Moleculares (CiQUS), Departamento de Química Orgánica, Universidade de Santiago de Compostela, 15782 Santiago de Compostela, Spain*

[b] *Centro Singular de Investigación en Química Biolóxica e Materiais Moleculares (CiQUS), Departamento de Química Inorgánica, Universidade de Santiago de Compostela, 15782 Santiago de Compostela, Spain. Email: miguel.vazquez.lopez@usc.es.*

[c] *Innopharma Drug Screening and Pharmacogenomics Platform. Center for Research in Molecular Medicine and Chronic Diseases (CiMUS). Department of Pharmacology, Pharmacy and Pharmaceutical Technology. Universidade de Santiago de Compostela, Santiago de Compostela, Spain.*

[d] *Health Research Institute of Santiago de Compostela, Santiago de Compostela, Spain*

[e] *Centro Singular de Investigación en Química Biolóxica e Materiais Moleculares (CiQUS), Departamento de Bioquímica e Bioloxía Molecular, Universidade de Santiago de Compostela, 15782 Santiago de Compostela, Spain*

[f] *Departament de Química, Universitat Autònoma de Barcelona, 08193, Bellaterra, Barcelona, Spain.*

[g] *Institut de Chimie (UMR 7177), University of Strasbourg – CNRS, 67081 Strasbourg, France.*

[h] *Institut Universitaire de France (IUF), 75231 Paris, France.*

[†] *These authors contributed equally*

Table of contents

Author contributions

1. Synthetic reagents

*2. Synthesis and purification of cyclopeptides **1** and **2***

*3. MALDI characterization of cyclopeptides **1** and **2***

4. Synthesis, purification and characterization of Ac-βAlaBpy-NH_2

*5. Cu(II) binding behavior of Ac-βAlaBpy-NH_2 and cyclopeptides **1** and **2** in water media*

6. Cytotoxicity studies

7. ICP studies

8. Electrochemical studies

9. ROS studies

10. Proposed most stable structures for the metallopeptide systems based in the experimental data

11. Molecular modeling studies

Author contributions

The following table describes the contribution of each author according to the CRediT taxonomy as described in A. Brand, L. Allen, M. Altman, M. Hlava, J. Scott, *Learn. Publ*. **2015**, *28*, 151–155. This taxonomy provides a detailed classification of the various roles performed by each author. The degree of contribution is coded as *lead* (black), *equal* (dark grey), or *supporting* (light grey).

	SB	DB	RLB	AxS	IS	DAG	JB	MIL	NBP	JMC	SM	GG	AIS	PF	MEV	MVL
Conceptualization*. Ideas, formulation of overarching research goals and aims.*									░			░		░	■	■
Methodology*. Development or design of methodology; creation of models.*			■	■		░		■	■	░		■		■	■	■
Validation*. Verification of the overall reproducibility of results and research outputs.*	░	░	░	░	░	░	░	■	■	░	░	■	░	■	■	■
Formal Analysis*. Application of formal techniques to analyze the data.*	░	░	░	■	░	░	░	■	■	░	░	■	░	■	■	■
Investigation*: synthesis of the compounds, metal binding.*	■	░			░											
Investigation*: ICP studies*		░	■			■										
Investigation*: Cytotoxic studies*	░	░					■									
Investigation*: Electrochemical studies*		░									■					
Investigation*: ROS studies*		░							■				■			
Investigation*: Molecular modeling studies*				■												
Resources*. Provision of reagents, materials, instrumentation, and analysis tools.*								■		■		■		■	■	■
Data Curation*. Management to annotate, scrub and maintain research data.*	░	░	░	░	░	░	░	░	░	░	░	░	░	░	░	■
Writing*: Original Draft. Preparation of the paper, specifically writing the initial draft.*		░										░		░		■
Writing*: Review & Editing. Critical review, commentary, or revision.*												░		░	■	■
Visualization*. Preparation, of the paper, specifically visualization &/or presentation.*															■	■
Supervision*. Oversight responsibility for the research planning & execution.*															░	■
Project Administration*. Management and coordination of the research.*															░	■
Funding Acquisition*. Acquisition of the financial support leading to this publication.*								░		░		░		░	■	■

1. Synthetic reagents

The solvents and reagents used in the synthesis of the Fmoc-βAla5Bpy-OH were obtained from *Fisher Chemical* (acetonitrile for HPLC, chloroform, toluene, DMF, methanol, NaOH), *Scharlau* (absolute ethanol), *Sigma Aldrich* (5,5'-dimethyl-2,2'-bipyridine, $KMnO_4$, $SOCl_2$, hydrazine monohydrate, xylene, DIPEA, 2-amino-2-methyl-1,3-propanediol (AMPD), $NaClO_4$, HCl 37%), *Panreac* (celite and $NaNO_2$). For the synthesis of the cyclopeptides, all the amino acids were provided by *Sigma Aldrich* and *IRIS Biotech GmbH* All the amino acids were used as their Fmoc protected derivatives. The resin employed for the SPPS was H-Rink-Amide *ChemMatrix* 35-100 mesh particle size from *Sigma Aldrich*. $CuCl_2{\cdot}2H_2O$ employed in the obtention of the Cu(II) metallopeptides was acquired to *Acros Organics.*

2. Synthesis and purification of cyclopeptides 1 and 2

2.1. Synthesis

The synthesis of cyclopeptides **1** and **2** was described in a previous paper.[1]

Cyclopeptides **1** and **2** were synthesized following standard solid phase peptide synthesis (SPPS) procedures and using a chlorotrityl resin. Arginines were coupled, in 5-fold excess (vs. mmol of resin load), by using O-(benzotriazol-1-yl)-N,N,N',N'-tetramethyluronium hexafluorophosphate (HBTU) as activating agent. Fmoc-βAlaBpy-OH was coupled in 3-fold excess by using 1-[bis(dimethylamino)methylene]-1H-1,2,3-triazolo-[4,5-b]pyridinium-3-oxide hexafluorophosphate (HATU) as activating agent. Couplings were conducted for 1 h. Deprotection of the temporal Fmoc protecting-group was performed with 20% piperidine in DMF for 20 min.

After the coupling of the complete amino acid sequences, the peptides, still with their arginine side chains protected, were cleaved from the resin using mild acidic conditions with diluted acetic acid (10% AcOH, 10% TFE, 80% CH_2Cl_2), which afforded the corresponding linear protected peptides. These intermediates were cyclized in solution using PyAOP as coupling agent, fully deprotected (in all cases) with a TFA cocktail (2.5%H_2O, 2.5%TIS, 5%CH_2Cl_2, 90%TFA).

2.2. Purification

Purification of cyclopeptides **1** and **2** was performed by preparative RP-HPLC with an *Agilent 1260 Infinity II series LC* with a *Agilent 1260* Binary Pump, using a Sunfire Prep C_{18} OBD (5 μm, 19 × 150 mm) reverse-phase column from *Waters*. Standard conditions for preparative RP-HPLC consisted of an isocratic regime during the first 2 min, followed by different linear gradients of solvent B during 30 min (A: water 0.1% TFA, B: acetonitrile 0.1% TFA). The gradients are adjusted for each compound undergoing purification, as appropriate. The purified samples were lyophilised. The cyclopeptide ligands were obtained as TFA salts of the protonated Arg residues.

[1] I. Salvadó, I. Gamba, J. Montenegro, J. Martínez-Costas, J. M. Brea, M. I. Loza, M. Vázquez López and M. E. Vázquez, *Chem. Commun.*, 2016, **52**, 11008–11011.

*a) Ciclopeptide **1***

52.1 mg obtained, 71% yield for a 0.05 mmol scale.

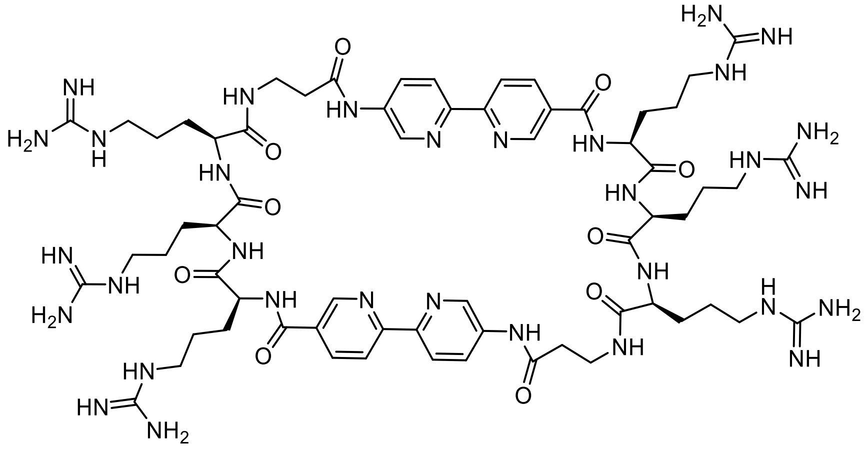

Scheme S1. *Chemical structure of cyclopeptide **1**.*

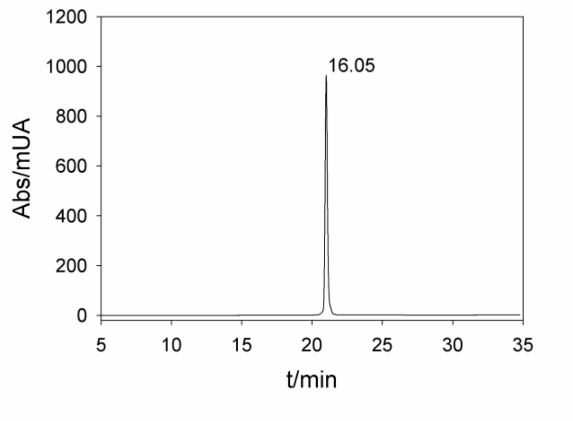

Figure S1. *Chromatogram of purified cyclopeptide **1**.*

*b) Ciclopeptide **2***

50.2 mg obtained, 68% yield for a 0.05 mmol scale.

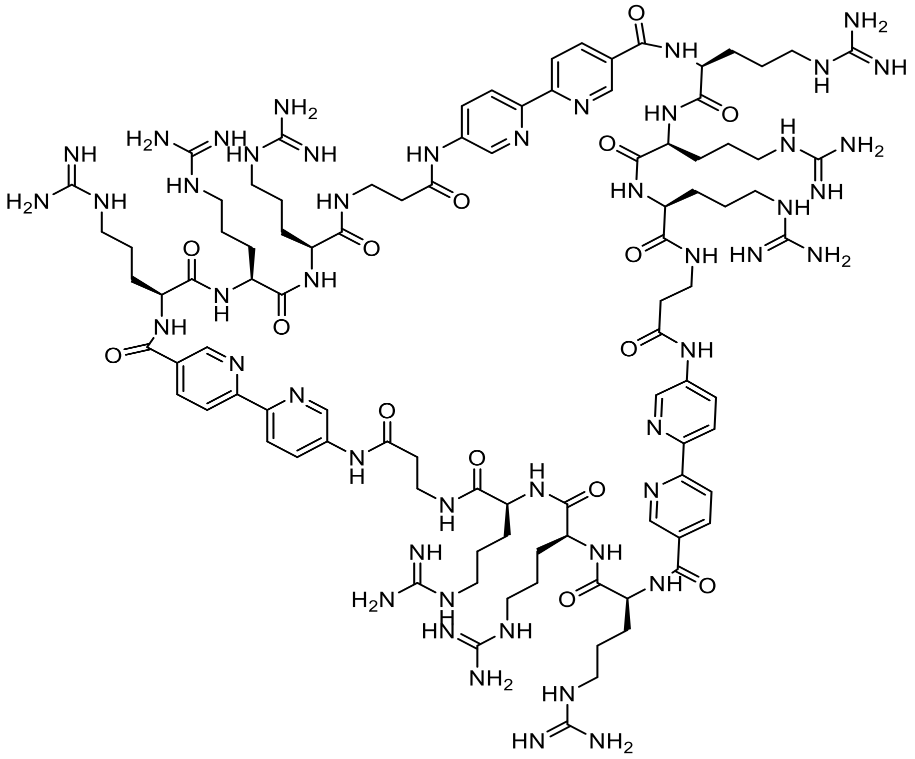

***Scheme S2.** Chemical structure of cyclopeptide **2**.*

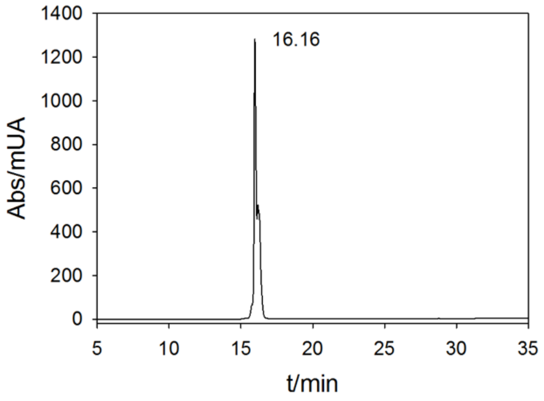

***Figure S2.** Chromatogram of purified cyclopeptide **2**.*

3. MALDI characterization of cyclopeptides 1 and 2

Cyclopeptides **1** and **2** were characterized by mass spectrometry (MALDI-TOF).

*a) Ciclopeptide **1***

MALDI-TOF: m/z calculated for $C_{64}H_{97}N_{32}O_{10}$: 1473.8, found $[M+H]^+$: 1473.8

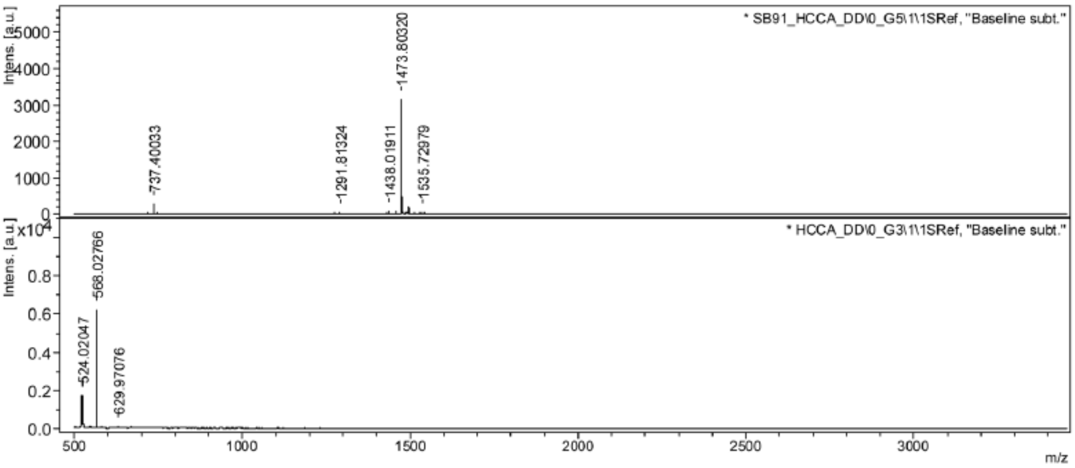

*b) Ciclopeptide **2***

MALDI-TOF: m/z calculated for $[M+H]^+$ $C_{96}H_{145}N_{48}O_{15}$: 2210.2, found: 2210.8

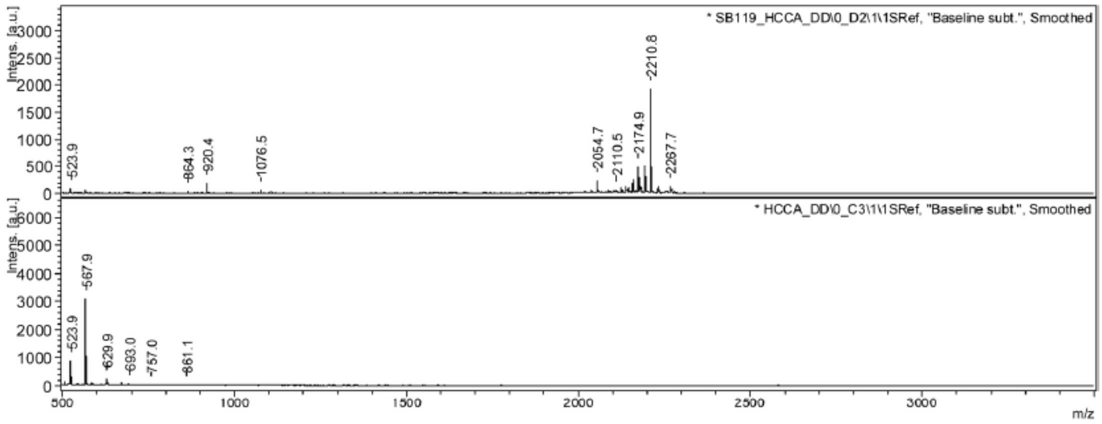

4. Synthesis, purification and characterization of Ac-βAlaBpy-NH_2

4.1. Synthesis

Ac-βAlaBpy-NH_2 (Scheme S1) was synthesized following the same procedure indicated in section 2 of this Supporting Information. After the coupling of the amino acid the temporal protecting group Fmoc was removed by treating the resin with a solution of 20% piperidine in DMF for 20 min. Then the free amine was acetylated by treating the resin with a solution of 0.8 mL Ac_2O, 2 mL DIEA/DMF (0.2 M) and 3.2 mL of DMF. The compound was cleavage from the resin using the usual TFA cocktail and purified by HPLC.

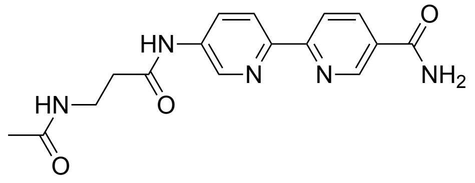

Scheme 3. *Structure of Ac-βAlaBpy-NH_2*

4.2. Purification

Purification of Ac-βAlaBpy-NH_2 was performed by preparative RP-HPLC with an *Agilent 1260 Infinity II series LC* with a *Agilent 1260* Binary Pump, using a Sunfire Prep C_{18} OBD (5 μm, 19 × 150 mm) reverse-phase column from *Waters*. Standard conditions for preparative RP- HPLC consisted of an isocratic regime during the first 2 min, followed by different linear gradients of solvent B during 30 min (A: water 0.1% TFA, B: acetonitrile 0.1% TFA). The gradients are adjusted for each compound undergoing purification, as appropriate.

4.3. Characterization

The analysis of compounds was performed by analytical UHPLC-MS with an *Agilent 1260 Infinity II series LC/MS* using a SB C18 (1.8 μm, 2.1 × 50mm) analytical column from *Phenomenex*. Standard conditions for analytical UHPLC consisted of a linear gradient from 5% to 95% of solvent B in 12 min at a flow rate of 0.350 mL/min (A: water 0.1% TFA, B: acetonitrile 0.1% TFA). Compounds were detected by UV absorption at 222, 280 and 310 nm. Electrospray Ionization Mass Spectrometry (ESI/MS) was performed with an *Agilent 6120 Quadrupole LC/MS* model in positive scan mode using direct injection of the purified compound solution into the MS detector.

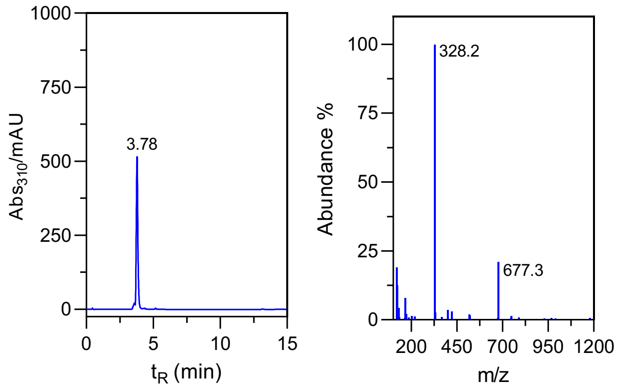

Figure S3. *Left, chromatogram of purified Ac-βAlaBpy-NH_2. Right, ESI mass spectra corresponding to the peak with t_R of 3.78 min.*

UHPLC-MS (ESI): (5-95% B t_R = 3.78 min) Calculated for $C_{16}H_{17}N_5O_3$ = 327.3; found $[M+H]^+$ = 328.2; $[M+2H]^{2+}$ = 165.1; $[2M+Na]^+$ = 677.3.

5. Cu(II) binding behavior of Ac-βAlaBpy-NH$_2$ and cyclopeptides 1 and 2 in water media

5.1. Mass spectrometry – MALDI-TOF

Matrix-assisted laser desorption/ ionization mass spectrometry (MALDI-MS) was performed with a *Bruker Autoflex* MALDI-TOF model in positive scan mode by direct irradiation of the matrix-adsorbed peptide. 4-HCCA (α-cyano-4-hydroxycinnamic acid) was the selected matrix for all these experiments.

a) ***1****/Cu^{II}*

MALDI-TOF: m/z calc for $[M+H]^+$ $C_{64}H_{97}N_{32}O_{10}$: 1473.8, found: 1473.9, calc. for $[M+Cu]^{2+}$ $C_{64}CuH_{96}N_{32}O_{10}$: 1537.3, found: 1535.8; $[M+2Cu]^{4+}$ calc. for $C_{64}Cu_2H_{96}N_{32}O_{10}$: 1600.8, found: 1599.7; calc. for $[M+3Cu]^{6+}$ $C_{64}Cu_3H_{96}N_{32}O_{10}$: 1664.3, found: 1661.7; calc. for $[M+4Cu]^{8+}$ $C_{64}Cu_4H_{96}N_{32}O_{10}$: 1727.8, found: 1725.5; calc. for $C_{128}Cu_4H_{192}N_{64}O_{22}$ $[2M+4Cu+2O]^{4+}$: 3231.6, found: 3233.9.

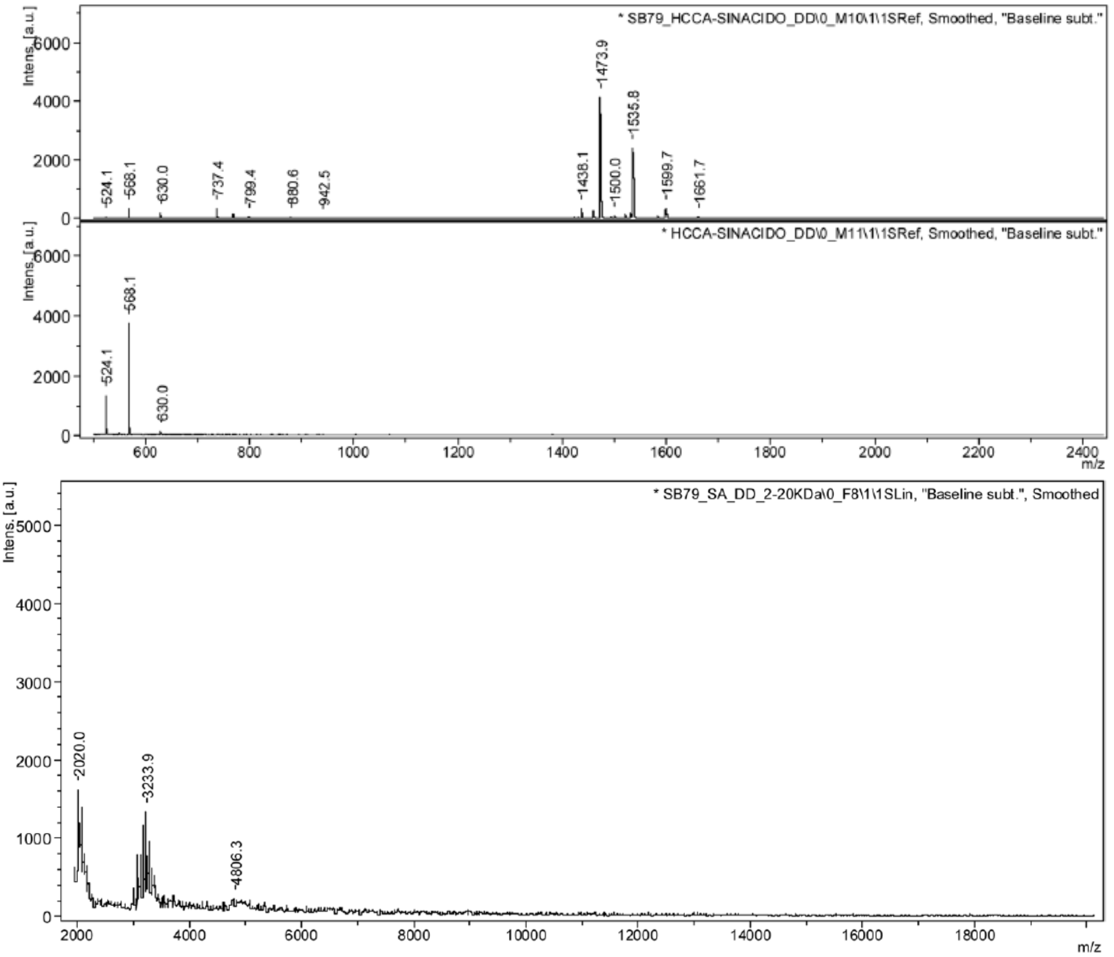

b) ***2****/Cu*^{II}

MALDI-TOF: m/z calc. for $[M+H]^{+}$ $C_{96}H_{145}N_{48}O_{15}$: 2210.2, found: 2211.3; calc. for $[M+Cu]^{2+}$ $C_{96}CuH_{144}N_{48}O_{15}$: 2272.7, found: 2273.2; calc. for $[M+2Cu]^{4+}$ $C_{96}Cu_2H_{144}N_{48}O_{15}$: 2336.2, found: 2336.1; calc. for $[M+3Cu]^{6+}$ $C_{96}Cu_3H_{144}N_{48}O_{15}$: 2399,7, found: 2398.0; calc. for $[M+4Cu]^{8+}$ $C_{96}Cu_4H_{144}N_{48}O_{15}$: 2463.2, found: 2461.0; calc. for $[2M+2Cu]^{4+}$ $C_{192}Cu_2H_{288}N_{96}O_{30}$: 4545.4, found: 4547.4; calc. for $[2M+3Cu]^{6+}$ $C_{192}Cu_3H_{288}N_{96}O_{30}$: 4608.9, found: 4610.6; calc. for $[2M+3Cu+2O]^{2+}$ $C_{192}Cu_3H_{288}N_{96}O_{32}$: 4640.9, found: 4645.1; calc. for $[2M+4Cu]^{8+}$ $C_{192}Cu_4H_{288}N_{96}O_{30}$: 4672.4, found: 4673.9; calc. for $[2M+4Cu+2O]^{4+}$ $C_{192}Cu_4H_{288}N_{96}O_{32}$: 4704.4, found: 4707.7.

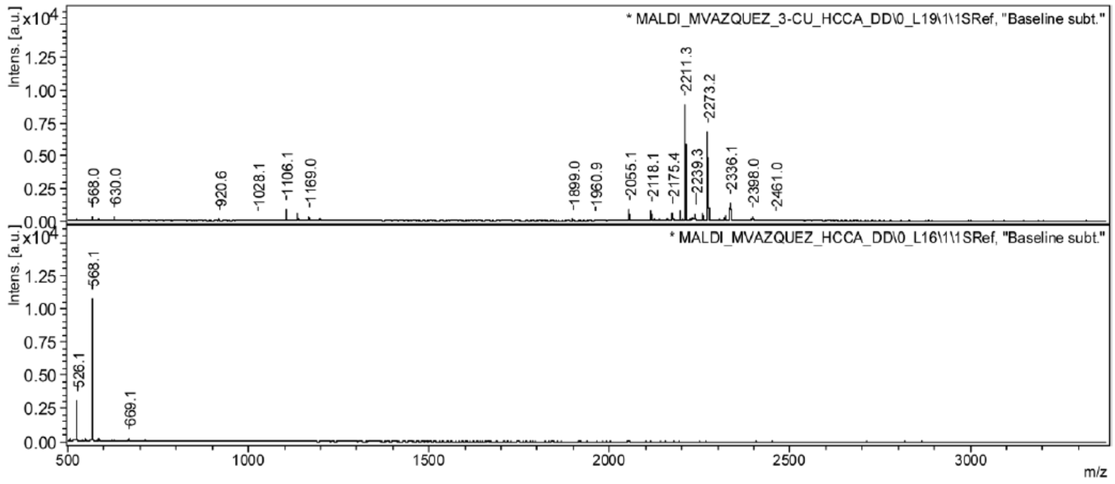

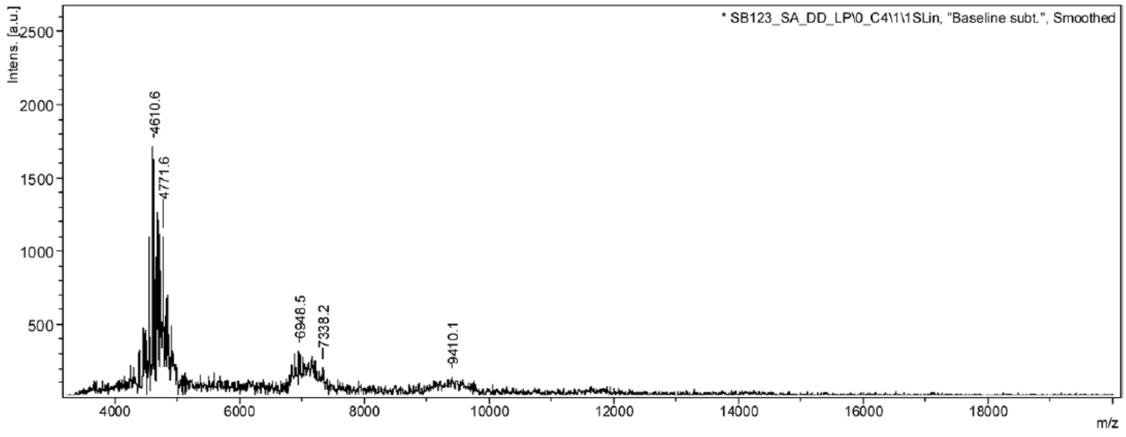

5.2. Fluorescence titration experiments

Luminescence experiments were made with a *Varian Cary Eclipse Fluorescence* spectrophotometer coupled to a *Cary Single Cell Peltier* accessory (*Agilent Technologies*) temperature controller. All measurements were made with a *Hellma* semi-micro cuvette (108F-QS) at 20 °C. The settings for these measurements were adapted depending on the studied system.

To a 2 μM solution of cyclopeptide **1** or **2** in phosphate buffer (1 mM, 10 mM NaCl, pH 6.5), aliquots of a stock solution of $CuCl_2 \cdot 2H_2O$ (as source of Cu^{II} ions) in water were added and the emission spectrum was recorded (λ_{ex}= 308 nm; λ_{em}= 410 nm) after each addition. The resulting titration profiles were fitted with Dynafit program to different binding modes to find out the stoichiometry and affinity of the cyclopeptide for the Cu^{II} ions. Titrations were made by triplicate.

Titrations were studied using the DynaFit software,[2] which adjusts the experimental data numerically, using a multidimensional Newton-Raphson algorithm to solve the nonlinear equations that describe the equilibrium. *Dynafit* requires plain text files called scripts that contains information about the chemical model underlying the experimental data, experimental values, such as starting concentrations of reactants, as well as information about location of the files.

A typical DynaFit script fitting the global 2:1 process

```
[task]
  data = equilibria
  task = fit

[mechanism]
  L + M + M <===> LM2    : Kdg dissoc

[constants]
  Kdg = 10.0  ?

[concentrations]
   L = 2.0  ; micro-M units

[responses]
   M   =     0.0
   L   =   100.0  ?
   LM2 =     1.0  ?

[data]
   variable  M
   monitor M, L, LM2
   offset auto ?
   directory ./labdata/cyclobetaAla/1/Flu/Cu
   sheet     1Cu.csv
   columns 2,3,4

;   plot logarithmic

[output]
   directory ./labdata/cyclobetaAla/1/Flu/Cu/out

[settings]
{Filter}
   AverageReplicates = y
{Output}
   XAxisLabel      = [Cu(II)]
   XAxisUnit       = micro-M
   YAxisLabel      = emission at 410 nm
   BlackBackground = n
   WriteTXT        = y

[end]
```

[2] a) P. Kuzmic, *Anal. Biochem.*, 1996, **237**, 260–273; b) P. Kuzmič, in *Methods in Enzymology*, Academic Press, 2009, vol. 467, pp. 247–280.

5.3. UV-Vis studies

UV measurements were made in a *Jasco V-770* spectrophotometer coupled to a *Jasco ETCR-762* temperature controller, using a standard *Hellma* semi-micro cuvette (119-004-10-40 QS) with a light path of 10 mm. Measurements were made at 20 °C. Acquisition parameters were: 200-700 nm range; scan speed of 1000 nm/min; data interval 2.0 nm; UV/Vis bandwidth 2.0 nm; UV/Vis response 0.24 s; baseline corrected.

To a solution of Ac-βAlaBpy-NH_2 or cyclopeptides **1** or **2** in phosphate buffer (1 mM, 10 mM NaCl, pH 6.5), aliquots of a stock solution of $CuCl_2 \cdot 2H_2O$ (as source of Cu^{II} ions) in water were added and the absorption spectrum was recorded after each addition. Titrations were made by triplicate.

In a different experiment, to a solution of cyclopeptides **1** or **2** in phosphate buffer (1 mM, 10 mM NaCl, pH 6.5), a specific volume of a stock solution of $CuCl_2{\cdot}2H_2O$ in water was added in such a way that metal saturation conditions were reached according to the stoichiometric calculations based on the fluorescence studies. The UV-Vis spectra were recorded before and after the addition of the Cu^{II} ions.

The extinction coefficients of the peptides are the following:

Ac-βAlaBpy-NH_2: ε= 28283 $M^{-1}cm^{-1}$

Cyclopeptide **1**: ε= 56566 $M^{-1}cm^{-1}$

Cyclopeptide **2**: ε= 84849 $M^{-1}cm^{-1}$

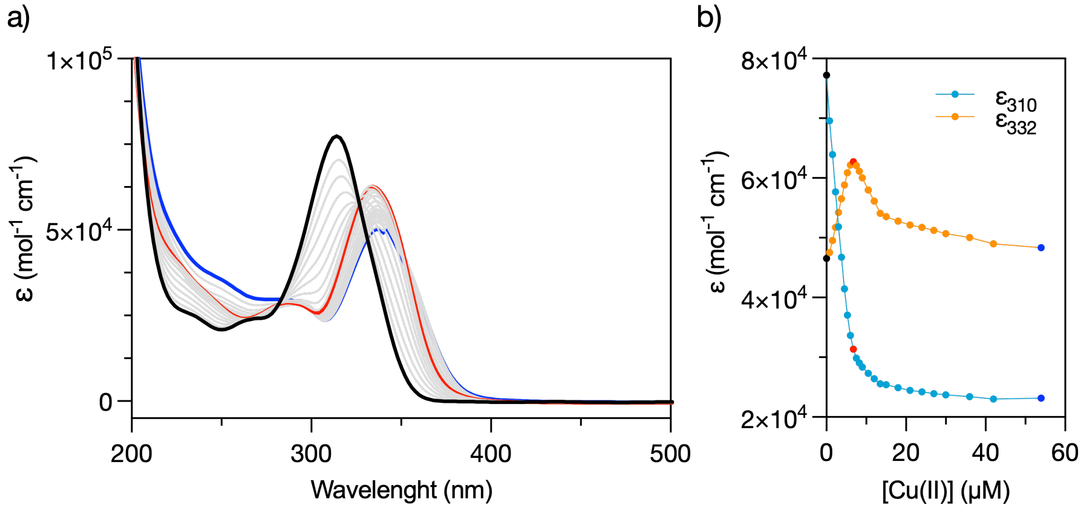

Figure S4. *a) UV-vis titration of a 6 μM solution of cyclopeptide* ***1*** *in phosphate buffer (1 mM, 10 mM NaCl, pH= 6.5) with a stock solution of Cu(II) ions. The black line belongs to the UV-vis spectra of the free cyclopeptide ligand, the red line belongs to the first coordinative process, that is, the formation of the 1:1 (L:M) specie (1.1 eq.* Cu^{II}*), and the blue line is the final spectrum (9 eq.* Cu^{II}*). b) Profiles of the titrations at 310 and 332 nm, highlighting the points corresponding to the black, red and blue spectra indicated in section a), with their corresponding colours.*

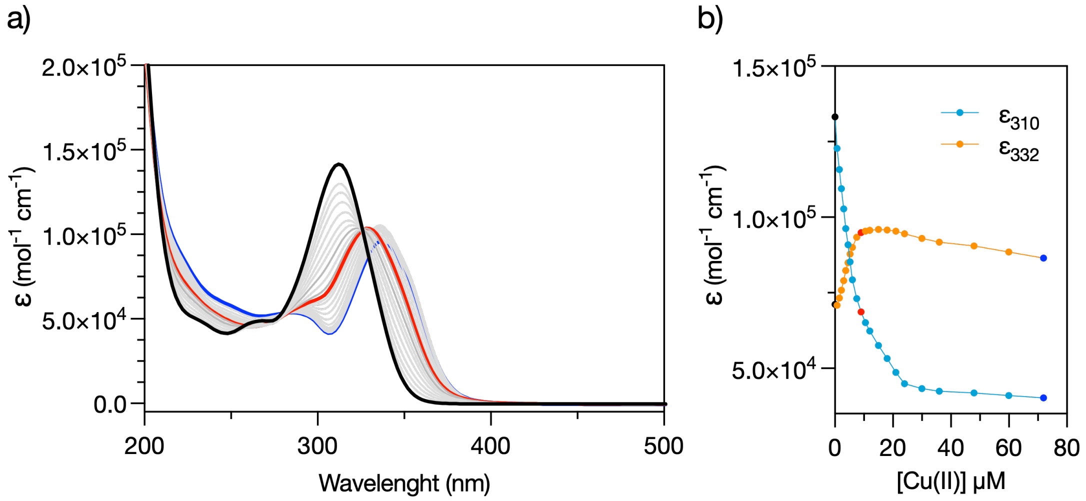

Figure S5. *a) UV-vis titration of a 6 μM solution of cyclopeptide* ***2*** *in phosphate buffer (1 mM, 10 mM NaCl, pH= 6.5) with a stock solution of Cu(II) ions. The black line belongs to the UV-vis spectra of the free cyclopeptide ligand, the red line belongs to the first coordinative process, that is, the formation of the 1:1 (L:M) specie (1.2 eq. Cu^{II}), and the blue line is the final spectrum (12 eq. Cu^{II}). b) Profiles of the titrations at 310 and 332 nm, highlighting the points corresponding to the black, red and blue spectra indicated in section a), with their corresponding colours.*

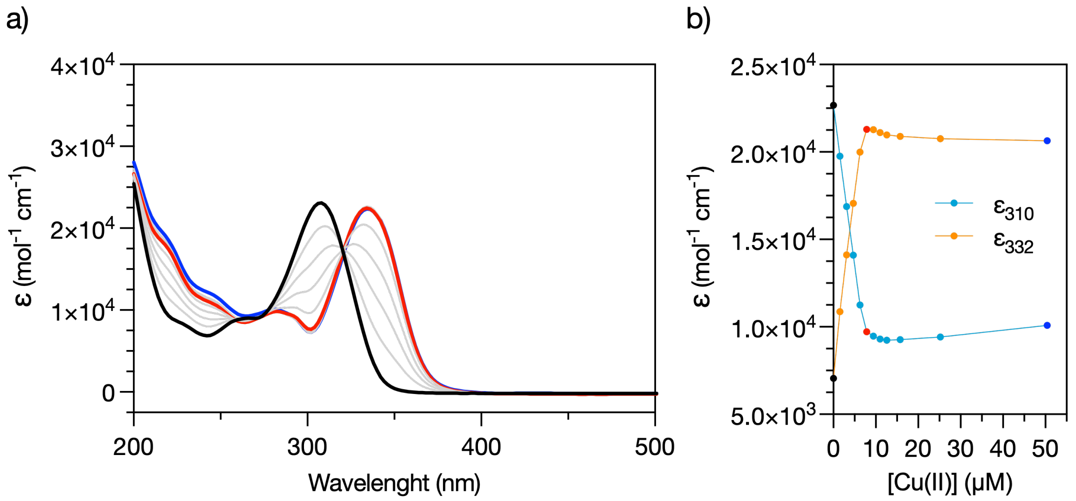

Figure S6. *a) UV-vis titration of a 12 μM solution of Ac-βAlaBpy-NH_2 in phosphate buffer (1 mM, 10 mM NaCl, pH= 6.5) with a stock solution of Cu(II) ions. The black line belongs to the UV-vis spectra of the free peptide ligand, the red line belongs to the end of the coordinative process, that is, the formation of the 2:1 (L:M) specie (0.65 eq. Cu^{II}), and the blue line is the final spectrum (4.1 eq. Cu^{II}). b) Profiles of the titrations at 310 and 332 nm, highlighting the points corresponding to the black, red and blue spectra indicated in section a), with their corresponding colors.*

5.4. Circular dichroism

Circular dichroism measurements were made with a *Jasco J-715* coupled to a *Neslab RTE-111* thermostated water bath, using a *Hellma* 100-QS cuvette (2 mm light pass). Scan speed was 200 nm/min and the obtained spectra are the mean of three accumulations.

To a 10 μM solution of the cyclopeptide **1** or **2** in phosphate buffer (1 mM, 10 mM NaCl, pH 6.5), a specific volume of a stock solution of $CuCl_2 \cdot 2H_2O$ in water was added in such a way that metal saturation conditions were reached according to the stoichiometric calculations based on the fluorescence studies. The CD spectra of the cyclopeptides were recorded before and after the addition of the Cu(II) ions.

5.5. EPR studies

EPR experiments were performed in a Bruker EMX instrument at a microwave frequency of 9.32 GHz (X-band), at room temperature. The experiments were recorded at a microwave power of 0.6 mW, modulation frequency of 100 kHz and modulation amplitude of 1 G. The solution with the **1**/Cu(II) metallopeptide system (200 μM of cyclopeptide **1** and 3 eq. of Cu^{II}) ions in phosphate buffer (1 mM, 10 mM NaCl, pH 7.0) were loaded into capillary EPR quartz tubes for the experiments.

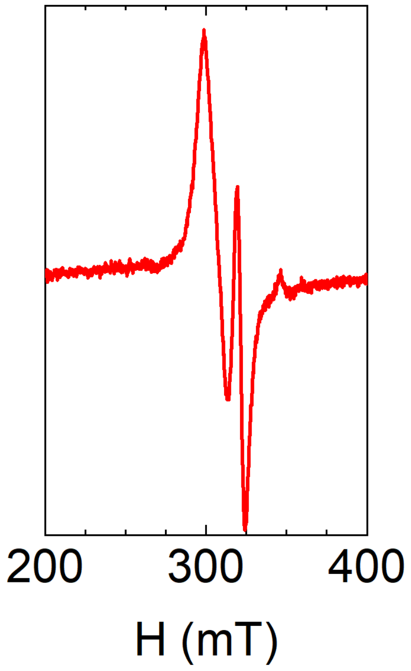

Figure S7. *Room temperature (300 K) EPR spectrum of **1**/Cu(II) metallopeptide system (200 uM of cyclopeptide **1** and 3 eq. of Cu^{II}) in phosphate buffer (1 mM, 10 mM NaCl, pH 7.0). The values for g// vary between 2.21 and 2.26 and for g⊥ between 2.05 and 2.1.*

6. Cytotoxicity studies

6.1. General method: cell lines and grown conditions

Non-small lung carcinoma NCI-H460, human colon carcinoma HCT-116, human glioma SF-268 and doxorubicin-resistance human mamma carcinoma NCI/ADR-RES cell lines, as well as Eagle's Minimal Essential Medium (EMEM) and RPMI1640 were purchased from the American Tissue Culture Collection (ATCC). Frozen cells were thawed in a 75 cm^2 cell culture flask in RPMI supplemented with 2 mM L-glutamine and 10% FBS and maintained at 37 °C in a 5% CO_2 atmosphere.

6.2. Cytotoxicity assay

The inhibition of cell proliferation induced by the samples [**1**/Cu(II), 1 eq. cyclopeptide **1**/3 eq. of Cu(II); **2**/Cu(II), 1 eq. cyclopeptide **2**/5 eq. of Cu(II)], was carried out by using MTT method for HCT-116, NCI/ADR-RES and NCI-H460 cell lines. SF-268 cell growth inhibition induced by the samples was assessed using a system based on crystal violet cell staining and subsequent acetylation. Cells were seeded in a 96-well microplate (5000 cells/well for HCT-116, 10000 cells/well for SF-268 and 15000 cells/well for NCI/ADR-RES and NCI-H460 cell lines) in 100 µl of growth medium and maintained at 37 °C for 24 h.

a) MTT method

Then growth medium was replaced by fresh medium containing different concentrations of the samples to be assayed and maintained at 37 °C in a 5% CO_2 atmosphere for 48 h. After this time, 10 µL MTT (5 mg/ml in PBS) were added to each well and maintained for 4 h at 37 °C in a 5% CO_2 atmosphere. Then 100 µL of 10% SDS in 0.01 M HCl were added to each well an incubated for 12-14 h under the same experimental conditions. Absorbance due to formation was detected in *a Tecan infinity M1000 Pro* reader using a wavelength of 595 nm. All assays were performed with triplicate points.

b) Crystal violet cell staining

The samples dissolved in water were added, maintaining the same proportion of water in each well (10%). After 72 h (at 37°C and in an atmosphere of 5% CO_2/95% air), the cells were fixed to the plate with 10 µL of 11% glutaraldehyde solution, kept under agitation at room temperature, for 15 minutes. Once fixed, the medium was removed and the cells were washed 3-4 times with distilled water, followed by staining of the cells with 100 µL of a 0.1% crystal violet solution. This solution was prepared by diluting 0.1g of crystal violet in 100 mL of buffer composed of 200 mM phosphoric acid, 200 mM formic acid and 200 mM 2-(N-morpholino)-ethane sulphonic acid (MES) at pH 6. The plate was kept shaking at room temperature for 15 minutes. The dye was removed, washed 3-4 times with distilled water and dried. 100 µL of 10% acetic acid was then added and kept under agitation for 15 minutes, at room temperature, so that the staining was homogeneous in all wells. Finally, an absorbance reading of the plates was performed at a wavelength of 595 nm (*Tecan M1000 Infinite Pro*). The assay was performed with triplicate points.

6.3. Data analysis

Data were expressed as the growth inhibition percentage calculated in basis on the equation: % inhibition= 100 – [(AO × 100) / AT]. Where AT is the measured absorbance in wells containing compounds and AO is the absorbance measured in blank wells (cells with medium and vehicle). The inhibitory potency of compounds was calculated by constructing concentration-% growth inhibition curves, and extrapolating IC_{50} values (concentration of compound that inhibits cells growth in a 50 %) from these curves. Curves were constructed by using *GraphPad Prism* software V2.01 (*GraphPad Inc*).

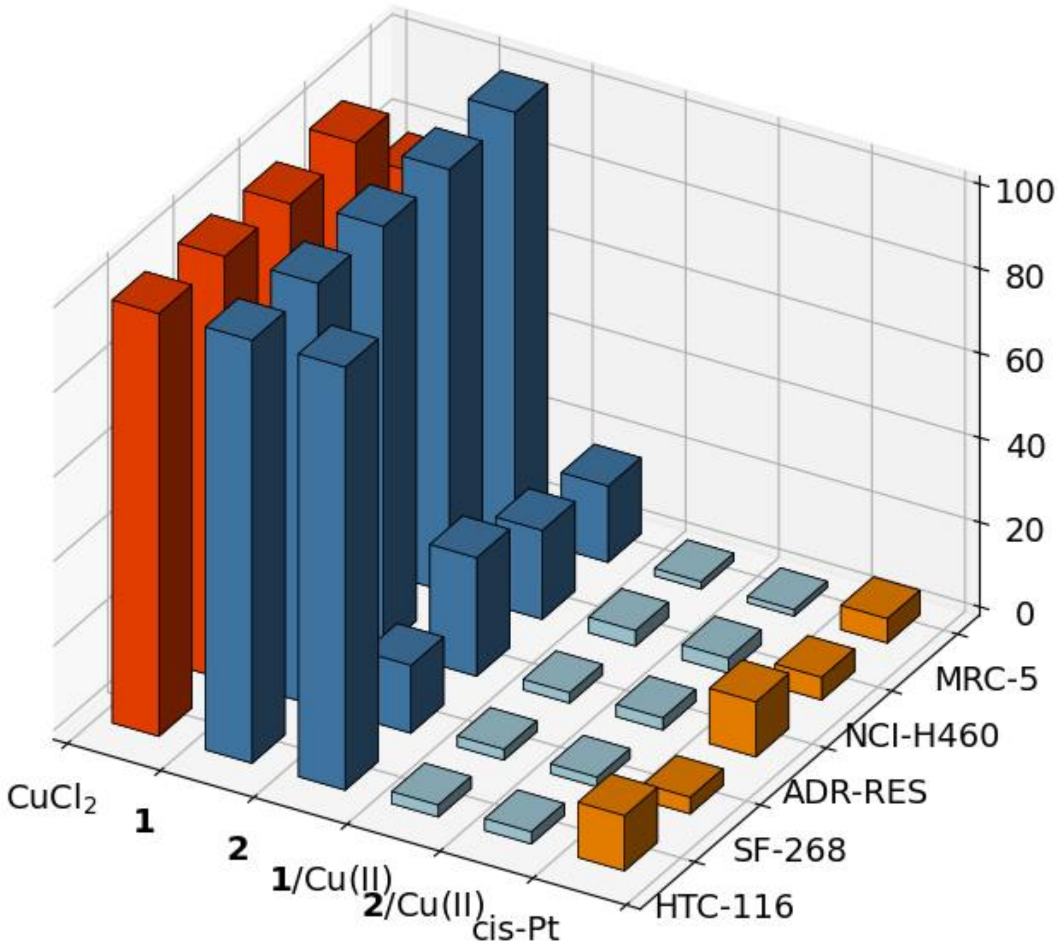

Figure S8. *IC_{50} values (μM) of cyclopeptides **1** and **2** (in dark blue) and their Cu(II) metallopeptide systems, **1**/Cu(II) and **2**/Cu(II) (in light blue). The activity of $CuCl_2.2H_2O$ (red) and cisplatin (dark yellow) were also measured for comparison (see Table 1 in the manuscript for full data). Incubation time in the presence of the metallopeptide systems was 48 hours for all cell lines except SF-268, which was 72 hours.*

7. ICP-MS studies

7.1. General methods and materials

ICP Measurements were conducted employing an *Agilent 7900* ICP-MS system (*Agilent Technologies, Tokyo, Japan*). The sample introduction system was comprised of an autosampler, a double-pass spray chamber with Peltier system, a glass concentric nebulizer (*MicroMist low-flow nebulizer*, *Glass Expansion*, *West Melbourne*, *Australia*), and a quartz torch. Quantification of copper concentrations was achieved using Agilent ICP-MS MassHunter 5.1, (*Version D.01.01 Agilent Technologies, Tokyo, Japan*). The parameters for the ICP-MS device were as follows: RF Power, 1550 W; Sample Depth, 8; Carrier Gas, 1.1 L/min; Nebulizer Pump 0.1 rps; S/C Temp, 2 °C.

Roswell Park Memorial Institute 1640 medium (RPMI), fetal bovine serum (FBS), penicillin, and streptomycin were purchased from Invitrogen Thermo Fisher.

7.2. Cell culture

Ovarian tumour NCI/ADR-RES cells, obtained from the European Collection of Authenticated Cell Cultures (ECACC), were cultured at 37 °C in a 5% CO_2 atmosphere in RPMI medium containing 10% FBS and supplemented with 50 U/mL penicillin and 50 U/mL streptomycin, in 75 cm^2 cell culture flasks. All cell experiments were performed at 37 °C in a 5% CO_2 atmosphere with this modified RPMI, referred as “medium” or “culture medium”.

7.3. Internalization studies

NCI/ADR-RES cells were seeded in 100 mm cell culture dishes (Thermo Scientific Nunc) at a density of 2×10^5 cells/mL in 1 mL of medium and incubated for 24 hours at 37 °C in 5% CO_2. The culture medium was then replaced with 3 mL of fresh medium containing 1 μM of the compound [Cu(**II**) ($CuCl_2{\cdot}2H_2O$), cyclopeptide (**1** or **2**), or metallopeptide system [**1**/Cu(**II**) or **2**/Cu(**II**); 1 eq. cyclopeptide + 1 eq. Cu(**II**)], followed by an additional 48-hour incubation at 37 °C in 5% CO_2. After incubation, the medium was removed, and cells were washed three times with PBS (6 mL per wash) before lysis in 3 mL of 70% HNO_3. Lysates were digested at 95 °C overnight, diluted with 1% HNO_3 to a final volume of 5 mL, and analyzed by ICP-MS. The Cu(**II**) content in the culture medium (3 mL) was also measured using the same technique, yielding a value of 41.46 ng. The experiments with the metallopeptide systems were conducted using equimolar amounts of cyclopeptide and Cu(**II**) to prevent an excess of free copper in the medium. All the experiments were conducted in triplicate.

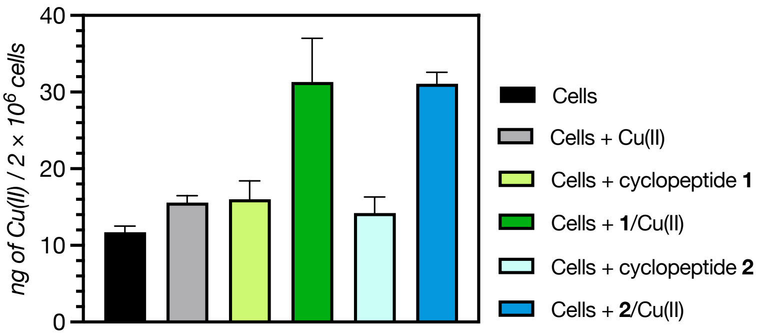

Figure S9. *ICP-MS results on the intracellular accumulation of Cu(II) ions in NCI/ADR-RES cells after 48 hours of incubation with culture medium containing 1 μM of the compound [Cu(II) ($CuCl_2{\cdot}2H_2O$), cyclopeptide (**1** or **2**), or metallopeptide system [**1**/Cu(II) or **2**/Cu(II); 1 eq. cyclopeptide + 1 eq. Cu(II))]. Data are mean +/- SEM for experimental repeated measurements. Label: Cu(II)= $CuCl_2.2H_2O$.*

8. Electrochemical studies

8.1. Electrochemical set up and conditions

To calibrate the working electrode and to evaluate the electrochemical setup used for the present studies, an aqueous solution of 50 mM AMPD/HCl pH 5 + 0.1 M $NaClO_4$ was used as electrolytic media. A three-electrode system was used; carbon vitreous electrode (diameter 1mm) was selected as working electrode, platinum as counter electrode and a saturated calomel (E = +0.241 V vs SHE at 25 °C) as reference electrode. All experiments were performed at room temperature.

8.2 Electrochemical characterization of $CuCl_2$ solution

To calibrate the working electrode and to evaluate the electrochemical setup used for the present studies, a fresh solution of $CuCl_2{\cdot}2H_2O$ in an aqueous solution of 50 mm AMPD/HCl pH 5 + 0.1 M $NaClO_4$ was first analyzed. Figure S2 shows that the electrochemical reduction of Cu(II) to Cu(I) is reversible (E^0=0.08 V vs. SCE) and relatively slow (ΔE_p=80 mV), which is in good agreement with previous data reported in the literature.[3] Cu(I) species also could be reduced into Cu^0 at E_{pc}=-0.34V (vs SCE) and oxidized into Cu(I) with a sharp wave at E_{pa}=0.01V (vs SCE). These potentials are used to discriminate free Cu(II) ions in the complex solutions.

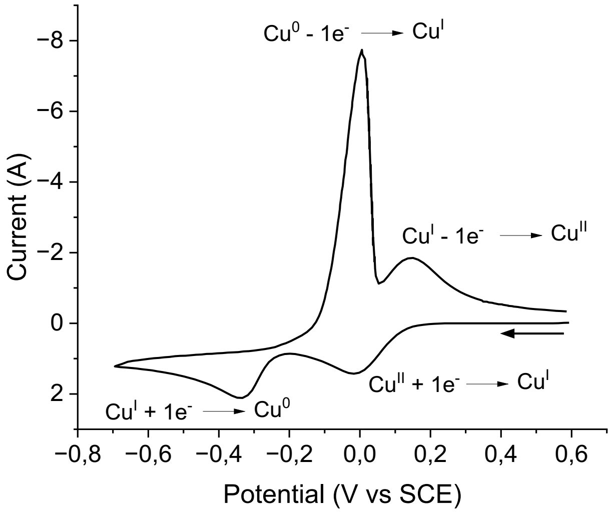

Figure S10. *CV of 1 mm $CuCl_2{\cdot}2H_2O$ in an aqueous solution of 50 mM AMPD/HCl pH 5 + 0.1 M $NaClO_4$ on a glassy carbon disk (diameter 1 mm). Scan rate: 0.3 $V{\cdot}s^{-1}$ (scan range -0.7/0.6 V). All measurements are referred to a SCE.*

8.3. Electrochemical characterization of cyclopeptides 1 and 2 in solution

Before starting the electrochemical studies of the cyclopeptides, we first analysed the electrochemical properties of 2,2'-bipyridine (Bpy), since it encloses a potentially active redox group. Therefore, a solution of Bpy 10 mM in a buffer solution of 50 mM AMPD/HCl pH 5 containing 0.1 M $NaClO_4$ (as supporting electrolyte), was analysed using CV. Typical CV is given in Figure S3e,f. A pseudo-reversible reduction peak at E_{pc}=-1.1 V (vs SCE) is observed.

Once established that Bpy is the electroactive synthon in the cyclopeptides, two different solutions of pure cyclopeptide **1** and **2** were analysed using CV. For both compounds, the CVs showed a cathodic pseudo-reversible wave at approximately -0.95 V versus SCE (Figure S3a-d). This reduction wave corresponds to the reduction of the Bpy residues, as can be clearly deduced from the peak potential (E_{pc}).

---

3 L. Sanz, J. Palma, E. Garcia-Quismondo, M. Anderson, *J. Power Sources*, **2013**, *224*, 278–284.

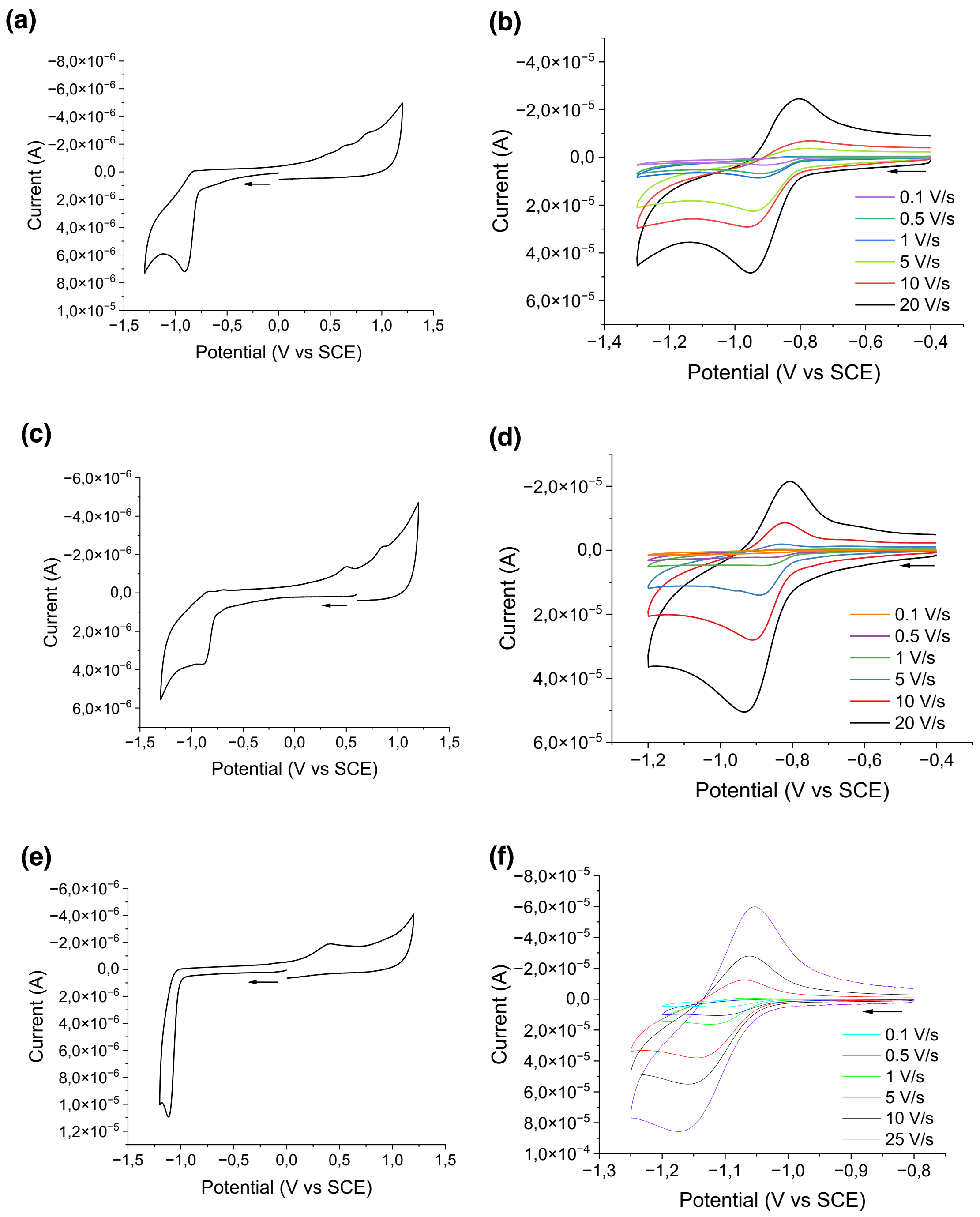

Figure S11. *Cyclic voltammetry of 1 mM cyclopeptides **1** and **2**, and 10 mM 2,2'-bipyridine in an aqueous solution of 50 mM AMPD/HCl pH 5 + 0.1 M $NaClO_4$, on a glassy carbon disk (diameter 1 mm). **(a)** Cyclopeptide **1** (v: 0.5 $V\cdot s^{-1}$). **(b)** Cyclopeptide **1** at different scan rates **(c)** Cyclopeptide **2** (v: 0.5 $V\cdot s^{-1}$). **(d)** Cyclopeptide **2** at different scan rates. **(e)** 2,2'-bipyridine (v: 0.5 $V\cdot s^{-1}$). **(f)** 2,2'-bipyridine at different scan rates. All measurements are referred to a SCE.*

9. ROS studies

9.1. Ascorbate studies

$CuCl_2 \cdot 2H_2O$, L(+)-ascorbic acid sodium salt (NaAscH), were purchased from *Sigma-Aldrich*. HEPES [4-(2-hydroxyethyl)-1-piperazineethanesulfonic acid] was purchased from *Alfa Aesar*.

$AscH^-$ oxidation ($[AscH^-]$ = 100 μM) was monitored by absorbance spectroscopy at λ_{max} = 265 nm (ε = 14,500 $M^{-1}cm^{-1}$). in 100 mM HEPES, pH 7.4, on a Clario Star plate reader at room temperature (25°C). Stock solutions of all the reactants were mixed inside a 96 wells transparent microplate (*Greiner UV-STAR® MICROPLATE*, 96 well, chimney form, flat bottom; dimensions: diameters 5 mm at the top and 4.38 mm at the bottom of chimney, high 11.5 mm,) with a final volume of 100 μL. $AscH^-$ autoxidation with O_2 was measured for 10 min, then the reaction was triggered by the addition of the preformed Cu(II)-complexes at 0.3 μM concentration.

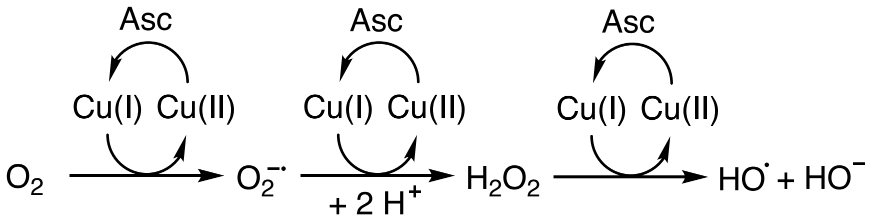

Scheme 4. *Oxidation of ascorbate by copper(I/II) complexes.*

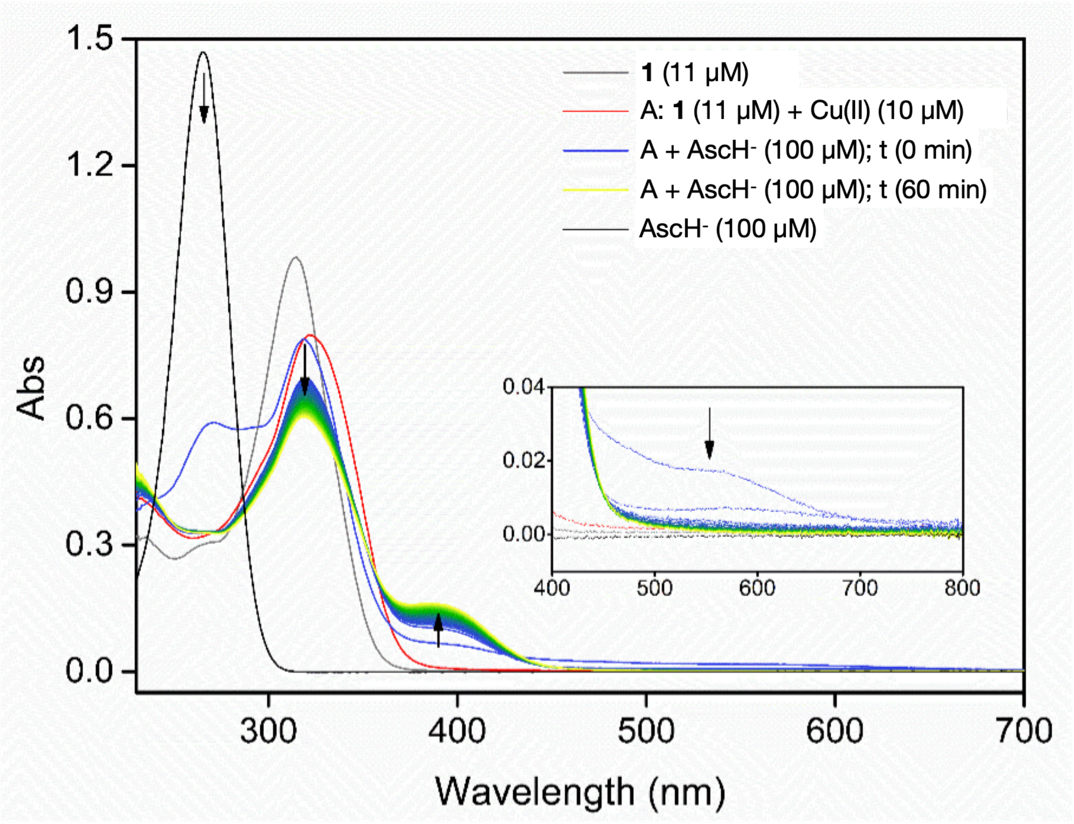

Figure S12. *Reactivity of a mixture of cyclopeptide **1** (11 μM) and Cu(II) ions (10 μM) with $AscH^-$ (100 μM) in 100 mM HEPES at pH 7.4, monitored by absorbance.*

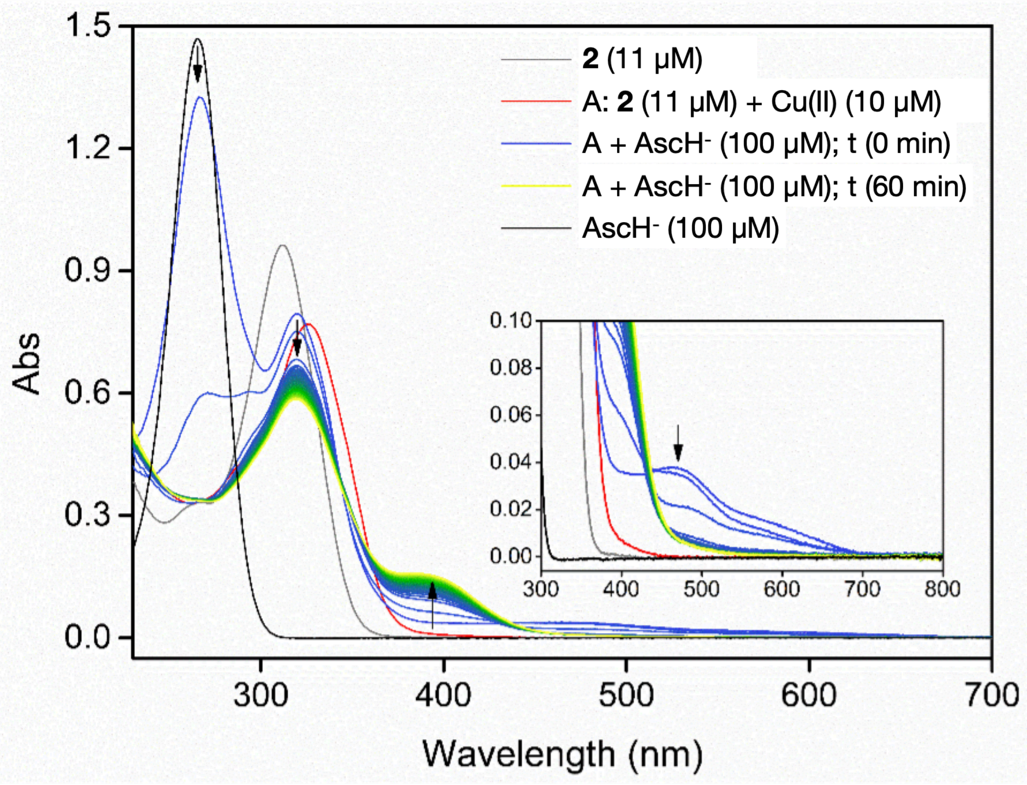

Figure S13. *Reactivity of a mixture of cyclopeptide **2** (11 μM) and Cu(II) ions (10 μM) with AscH*$^{-}$ *(100 μM) in 100 mM HEPES at pH 7.4, monitored by absorbance.*

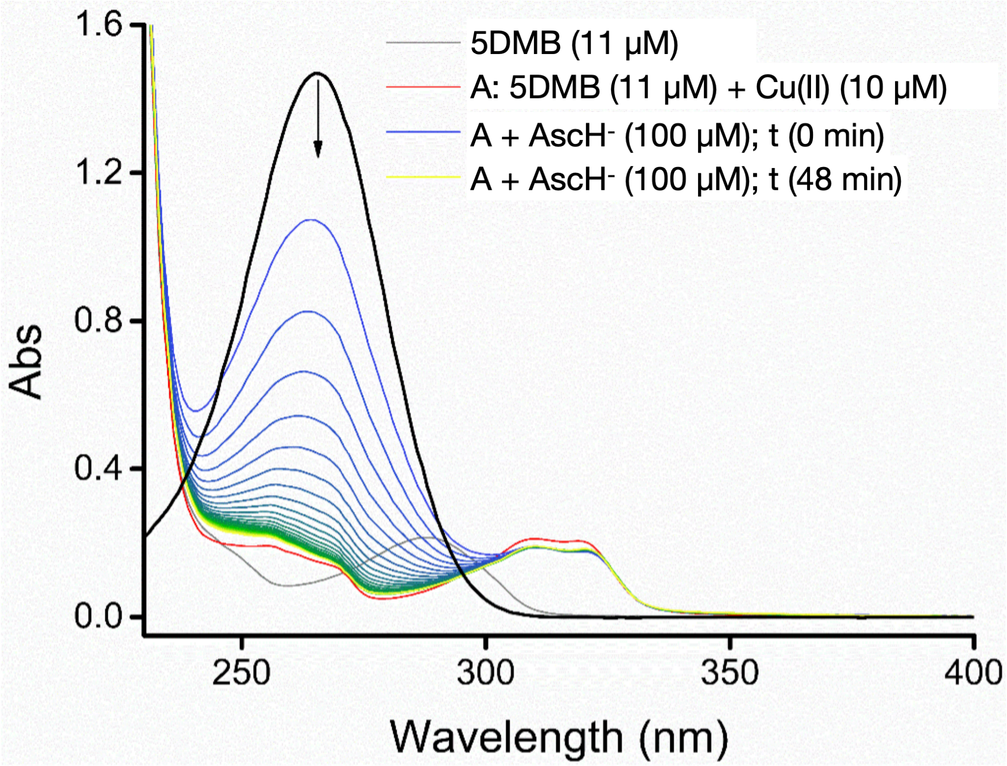

Figure S14. *Reactivity of a mixture of 5,5'-dimethyl bipyridine (5DMB) (11 μM) and Cu(II) ions (10 μM) with AscH*$^{-}$ *(100 μM) in 100 mM HEPES at pH 7.4, monitored by absorbance.*

9.2. Evaluation of intracellular ROS generation by the 1/Cu(II) metallopeptide system using ROS Assay Stain 1X

NCI/ADR-RES cells were seeded in a 96 well plate (Thermo Scientific 165305) at a density of 2,5 x 10^5 cells/ml. After 24 h of incubation at 37°C in 5% CO_2, culture media was replaced with 100 µl of ROS Assay Stain 1X (*Total Reactive Oxygen Species ROS Assay kit 520 nm*) and incubated at 37°C in 5% CO_2 for 1 hour. Ros Assay Stain was removed, and cells were washed with RPMI three times. Then, cells were incubated with 100 µM of Cu ($CuCl_2 \cdot 2H_2O$), cyclopeptide **1** or the **1**/Cu(II) metallopeptide system [1 eq. of cyclopeptide + 1 eq. of Cu(II)], and 125 µM de H_2O_2 and incubated at 37°C in 5% CO_2. The fluorescence was measured in a microplate reader every hour for the first 5 hours, and again at 24 hours after the addition of the compounds (λ_{ex}= 495 nm / λ_{em}= 520 nm). The experiment with the metallopeptide system was conducted using equimolar amounts of cyclopeptide **1** and Cu(II) to prevent an excess of free copper in the medium. All the experiments were conducted in triplicate.

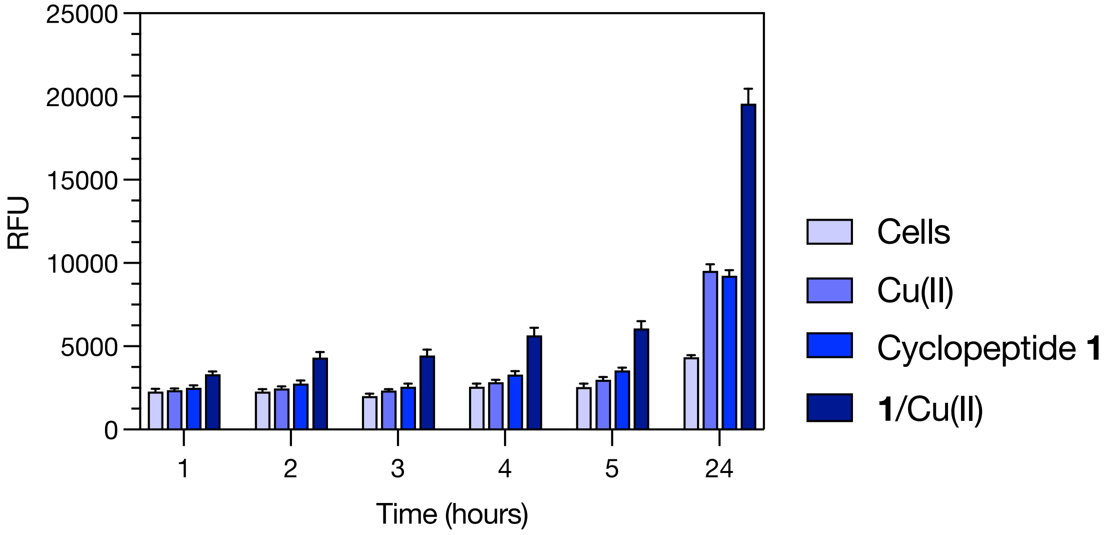

Figure S15. *Measurement of intracellular ROS levels in NCI/ADR-RES cells treated with Cu(II), cyclopeptide **1**, or the **1**/Cu(II) metallopeptide system. After 24 h incubation, cells were stained with ROS Assay Stain 1X and treated with 100 µM of Cu(II), cyclopeptide **1**, or **1**/Cu(II), along with 125 µM H_2O_2. Fluorescence (λ_{ex}= 495 nm / λ_{em}= 520 nm) was recorded every hour for the first 5 hours, and again at 24 hours after the addition of the compounds to assess intracellular ROS generation. Label: Cu(II)= $CuCl_2.2H_2O$.*

10. Proposed most stable structures for the metallopeptide systems based in the experimental data

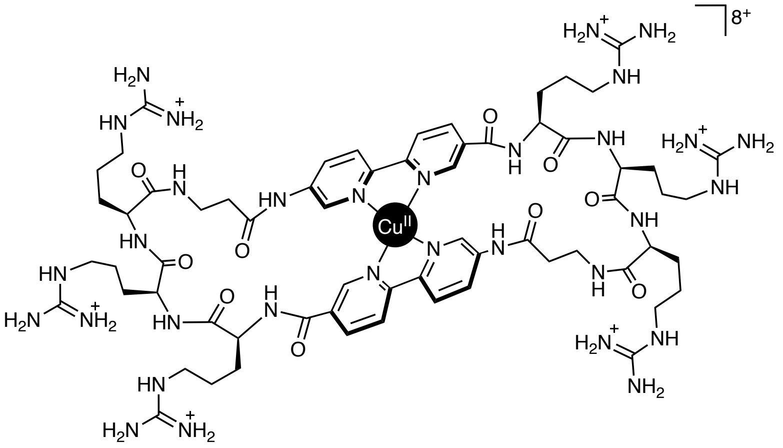

Figure S16. *Proposed structure of the most stable specie, and therefore the majority in solution during the biological studies performed, for the* ***1****/Cu(II) metallopeptide system, as suggested by the experimental data obtained. As Cu(II) is a d^9 system which undergo Jahn-Teller distortion, and taking into account previous studies in water with the complex $[Cu(Bpy)_2]^{2+}$, its coordination geometry in this metallopeptide specie is suggested to be distorted square planar.*

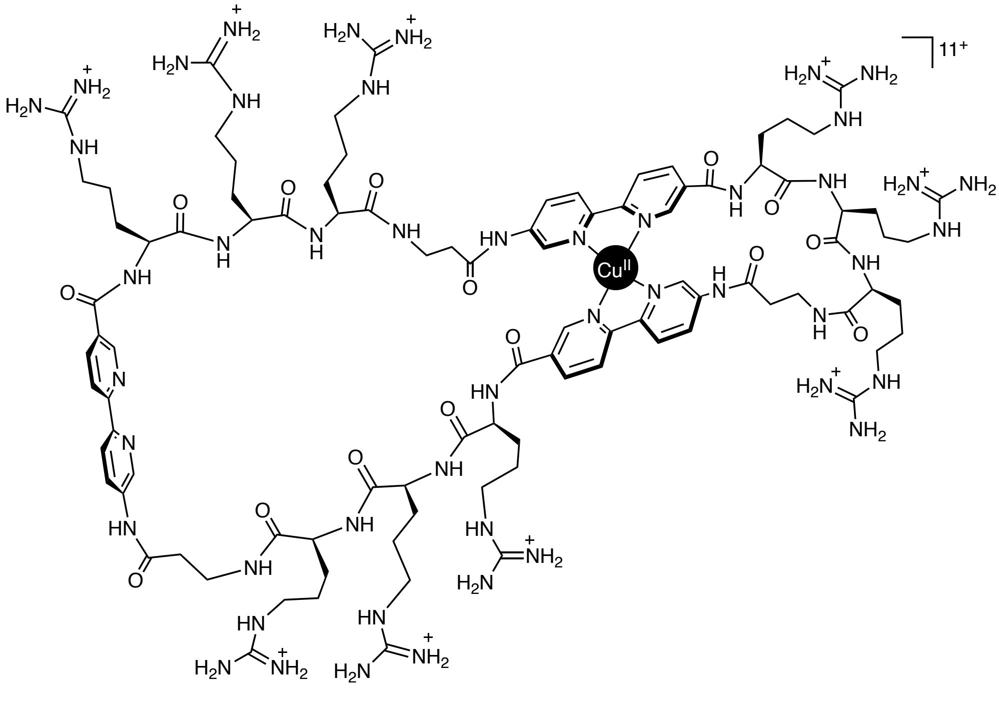

Figure S17. *Proposed structure of the most stable specie, and therefore the majority in solution during the biological studies performed, for the* ***2****/Cu(II) metallopeptide system, as suggested by the experimental data obtained. As Cu(II) is a d^9 system which undergo Jahn-Teller distortion, and taking into account previous studies in water with the complex $[Cu(Bpy)_2]^{2+}$, its coordination geometry in this metallopeptide specie is suggested to be distorted square planar. In the proposed structure, based on the experimental evidence described in this work, one bipyridine ligand remains uncoordinated. We acknowledge that this bipyridine unit could potentially bind to Cu(II) ions present in solution. However, in such a case, the Cu(II) center would have to complete its coordination sphere with water molecules, resulting in a highly labile and less thermodynamically stable coordination environment. For this reason, we have intentionally depicted it as vacant, emphasizing the coordination versatility inherent to cyclopeptide* ***2****.*

11. Molecular modeling studies

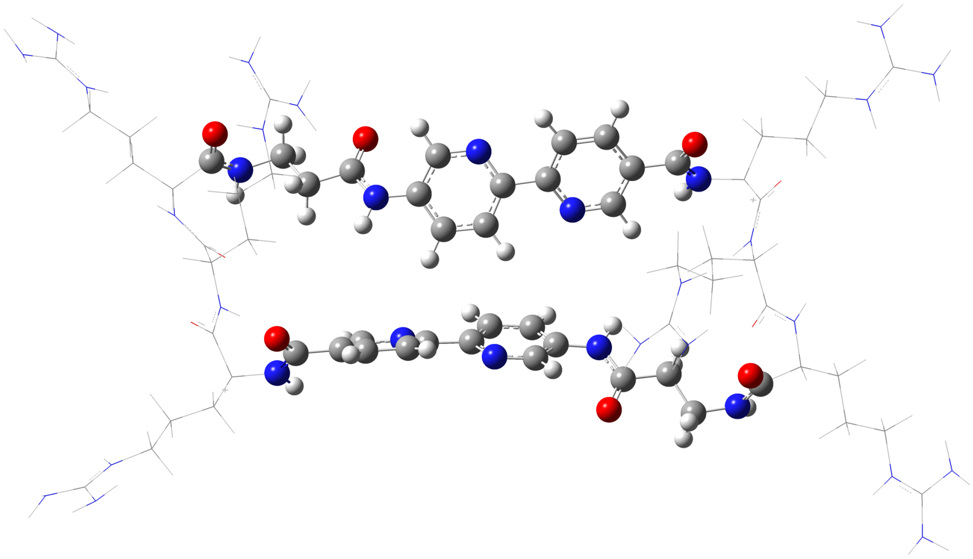

Figure S18. *Representation of cyclopeptide **1** after the ONIOM calculation. The arginine residues are shown in a wireframe model, while βAlaBpy residues are depicted using a ball-and-stick representation.*

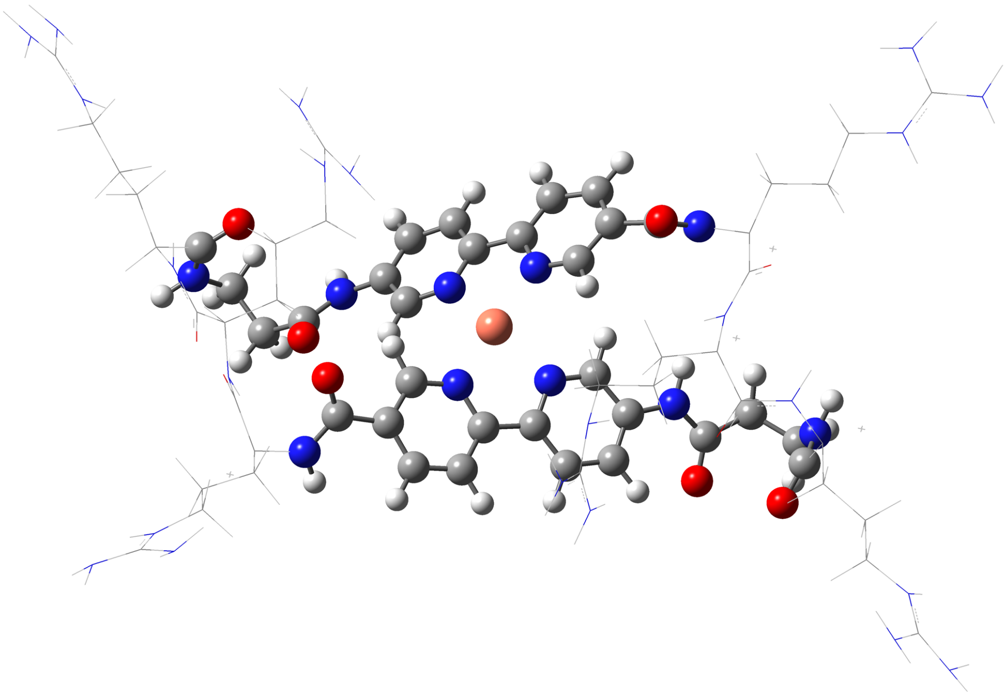

Figure S19. *Representation of the proposed most stable structure of the **1**/Cu(II) metallopeptide system after ONIOM calculation. Arginine residues are shown in a wireframe model, while βAlaBpy residues and the Cu(II) ion are depicted using a ball-and-stick representation.*
